# The sustainability of trade in wild plants—A data-integration approach tested on critically endangered *Nardostachys jatamansi*

**DOI:** 10.1093/pnasnexus/pgad328

**Published:** 2023-11-07

**Authors:** Carsten Smith-Hall, Dipesh Pyakurel, Henrik Meilby, Mariève Pouliot, Puspa L Ghimire, Suresh Ghimire, Sofia T Madsen, Yagya R Paneru, Bhishma P Subedi, Anastasiya Timoshyna, Thorsten Treue

**Affiliations:** Department of Food and Resource Economics, Faculty of Science, University of Copenhagen, Rolighedsvej 23, Frederiksberg 1958 C, Denmark; Resources Himalaya Foundation, Damkal Charkrapath Marg 10007, Lalitpur Metropolitan City-3, Lalitpur, Nepal; Department of Food and Resource Economics, Faculty of Science, University of Copenhagen, Rolighedsvej 23, Frederiksberg 1958 C, Denmark; Department of Food and Resource Economics, Faculty of Science, University of Copenhagen, Rolighedsvej 23, Frederiksberg 1958 C, Denmark; Asia Network for Sustainable Agriculture and Bioresources, 819/29 Bhimsengola Marg, Kathmandu Metropolitan City-31, Kathmandu, Nepal; Central Department of Botany, Tribhuvan University, Kirtipur Municipality-10, Kathmandu, Nepal; Department of Sustainability and Planning, University of Aalborg, A.C. Meyers Vænge 15, 2450 Copenhagen, Denmark; National Herbarium and Plant Laboratories, Satdobato-Godavari Rd, Godawari-3, Lalitpur, Nepal; Asia Network for Sustainable Agriculture and Bioresources, 819/29 Bhimsengola Marg, Kathmandu Metropolitan City-31, Kathmandu, Nepal; TRAFFIC International, Cambridge, David Attenborough Building, Pembroke St, Cambridge CB2 3QZ, UK; Department of Food and Resource Economics, Faculty of Science, University of Copenhagen, Rolighedsvej 23, Frederiksberg 1958 C, Denmark

**Keywords:** commercial harvesting, conservation policy, environmental products, Himalaya, illegal wildlife trade

## Abstract

While the demand for many products from wild-harvested plants is growing rapidly, the sustainability of the associated plant trade remains poorly understood and understudied. We integrate ecological and trade data to advance sustainability assessments, using the critically endangered *Nardostachys jatamansi* in Nepal to exemplify the approach and illustrate the conservation policy gains. Through spatial distribution modeling and structured interviews with traders, wholesalers, and processors, we upscale district-level trade data to provincial and national levels and compare traded amounts to three sustainable harvest scenarios derived from stock and yield data in published inventories and population ecology studies. We find increased trade levels and unsustainable harvesting focused in specific subnational geographical locations. Data reported in government records and to CITES did not reflect estimated trade levels and could not be used to assess sustainability. Our results suggest that changing harvesting practices to promote regeneration would allow country-wide higher levels of sustainable harvests, simultaneously promoting species conservation and continued trade of substantial economic importance to harvesters and downstream actors in the production network. The approach can be applied to other plant species, with indication that quick and low-cost proxies to species distribution modeling may provide acceptable sustainability estimates at aggregated spatial levels.

**Significance Statement**

Thousands of wild-harvested plant species are traded globally. For most species, there is scant standardized and valid data on harvested amounts or the status of plant populations, in particular at national level, and little is known about the sustainability of trade. We present a data-integration approach specifying what ecological and trade data is required to develop sustainability estimates. We exemplify how such data can be generated, integrated, and upscaled using the example of the critically endangered *Nardostachys jatamansi* harvested in and traded from Nepal. This also exemplifies how existing data can be better used. We identify subnational areas subject to unsustainable harvesting and discuss the policy insights and conservation potentials this approach offers.

## Introduction

Loss of biodiversity is a critical global problem threatening human well-being through disruption of ecosystems and loss of associated goods and services (1). An increased focus on the sustainable use of environmental resources is needed (2). Commercial harvesting is a primary driver of species' decline (3), but there is limited integration and upscaling of data to inform conservation interventions (4). A study of the trade in pangolins in Africa (5) collated local-scale market studies and argued that increases in prices and the number of juvenile or subadult animals killed indicated unsustainable hunting; a study of ephiphytic orchids in Mexico (6) combined domestic trade information with global population dynamics studies to illustrate the potential for sustainable harvesting of pseudobulbs. Acknowledging that sustainability assessments are influenced by many factors, including taxonomic group and study site characteristics (7), a generalized approach to data integration and upscaling for improved sustainability assessments is lacking.

Responding to calls for more focus on plants in wildlife trade and conservation studies (8), the low share of plants in harvest sustainability studies (7), the rarity of empirically based sustainability assessments (9), and recognizing the large scale and speed of change in the trade of many plant products (10, 11), the purpose is to propose a data-driven approach to advance sustainability assessments for traded wild-harvested plant species. We test it on the critically endangered alpine perennial *Nardostachys jatamansi* in Nepal, demonstrating that existing data can be used differently to achieve better-informed species conservation decisions.

### An analytical model for data integration and upscaling to advance sustainability assessments

**Fig. 1.**
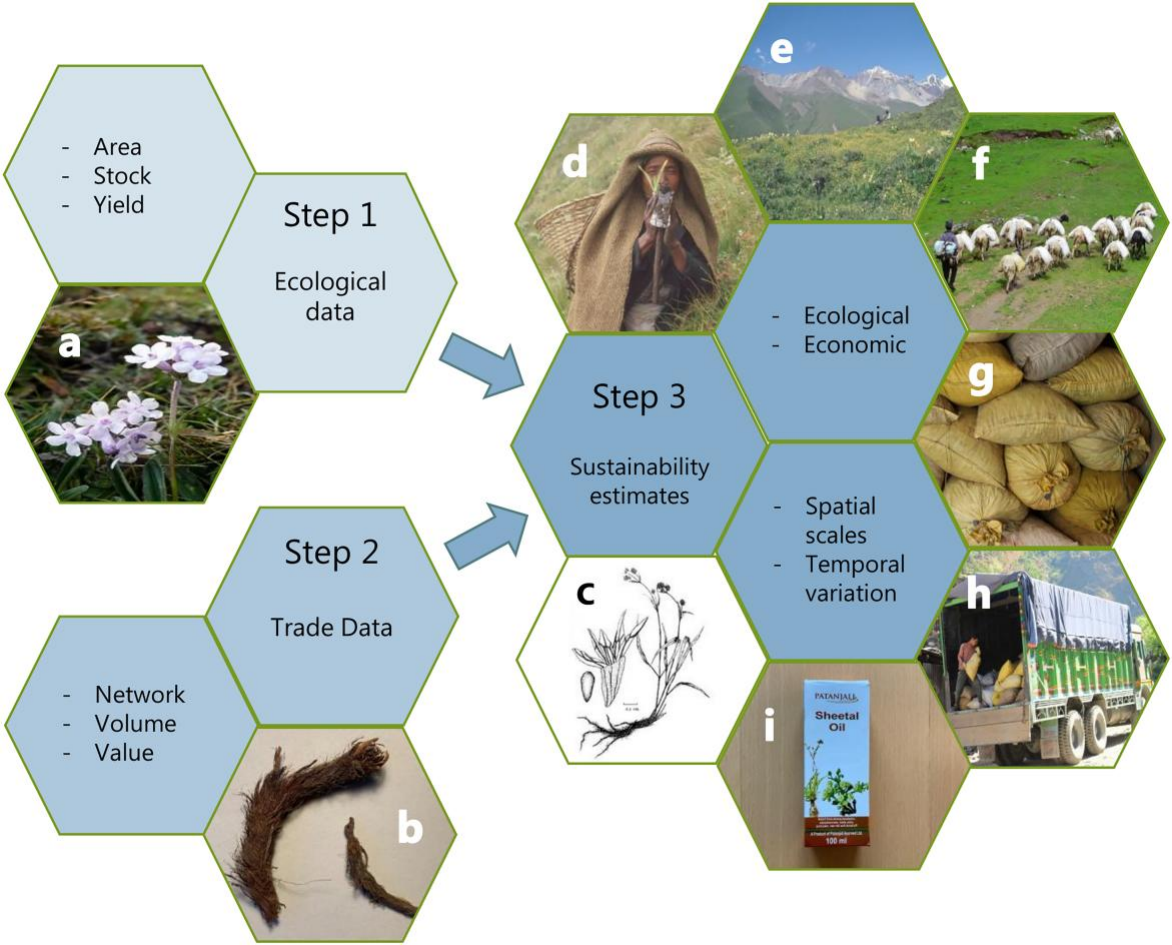
The data-driven three-step analytical framework for assessing the sustainability of wild-harvested commercial plant species. Input variables (Steps 1 and 2) and output dimensions (Step 3) are listed in the neighboring same-colored hexagons. Photos illustrate the example of spikenard (*Nardostachys jatamansi*) in Nepal: flowers (gracilis type, (a)); harvested air-dry rhizomes (b); sketch of seed, fruit, and whole plant with rhizome (c); harvester holding autumn specimen (d); alpine meadow harvesting area (e); sheep used for transport to road network (f); storage in traders' gunny sacks (g); loading of truck going to India (h); final consumer product (i). Credits: Suresh Ghimire (a); Carsten Smith-Hall (b, d, e, g, h, i); NC Shah, under a Creative Commons license CC-BY-SA-4.0 (c); Dipesh Pyakurel (f). Layout inspired by (11].

While approximately 374,000 plant species are known (12), only 2,500 have been domesticated to some degree and 250 fully, with their lifecycle dependent on human cultivation (13). As the number of traded species is huge, e.g. the Convention on International Trade in Endangered Species of Wild Fauna and Flora protects around 32,800 plant species against over-exploitation through international trade (14), most traded plant species are wild-harvested. We propose a data-driven three-step parsimonious approach to assess the sustainability of commercial wild-harvested plant species (Fig. 1). The first step is collecting basic ecological data on: (i) a population's distribution area (hectares), determined through modeling, (ii) the stock (number and weight of harvestable products) per unit area and site type, determined through inventories, and (iii) the yield (annual production of harvestable products) per unit area and site type, determined through yield or growth studies. The second step is collecting basic trade data on: (iv) production network (value chain) characteristics (location, function, and type of actors) in order to design data collection on (v) volume (kg) and (vi) value (USD per kg at specific points in the network). The third step is integrating the ecological and trade data to estimate sustainability (ecological and economic) at the relevant spatial (geographical or administrative) and temporal levels. While the model is simple, the challenge is to generate the required data—little information is available on wild-harvested plants, particularly for nontimber species. For instance, population ecology studies are available for only 1% (15) of the 300 medicinal plant, fungus, and lichen species traded in Nepal (16).

We exemplify the approach using *N. jatamansi* in Nepal, subject to increasing international attention (17–20) and traded in known production networks for high-altitude medicinal plants (15, 21–26): harvesters collect rhizomes in subalpine, alpine, and Trans-Himalayan vegetation types in the summer and autumn, and sell the air-dried products to village-based traders whose customers are larger traders in market centers such as district capitals. These organize transport and sell to central wholesalers in cities, from where the products are sold to domestic processors or exported to regional wholesalers in India and China. Temporal changes are analyzed using two comparable data sets (the case years of 1997–1998 and 2014–2015, the only years for which comprehensive district-level trade data is available). Sustainability issues have been discussed for decades, but hard data to support/refute claims of (un)sustainability for any species is minimal (15, 27). Here, we take an operational and pragmatic data-driven approach to species-level ecological sustainability, narrowly defined as occurring when wild harvesting does not exceed the annual growth per unit area.

The variables in Fig. 1 are species-specific. We focus on *N. jatamansi* (Supplementary Information 1) because it is: (i) in a monotypic genus, (ii) vulnerable due to high and rising prices, limited distribution, small population size, and high habitat specificity (16), (iii) exported to the Middle East and Europe for millennia (28), (iv) traded in high amounts with 82% of the annual global production (29) and 99% of legal international trade (Supplementary Information 11) from Nepal, (v) exclusively wild-harvested and economically important to rural households (21), and (vi) traded in dynamic cross-border production networks (22, 23) influenced by major ongoing changes, such as the Belt and Road Initiative (11).

## Results

We present ecological and trade data, estimate sustainable harvest levels using three different sustainability scenarios, and compare findings with official government records and CITES trade data.

### Step 1—Ecological data: area, stock, and yield estimates

**Fig. 2.**
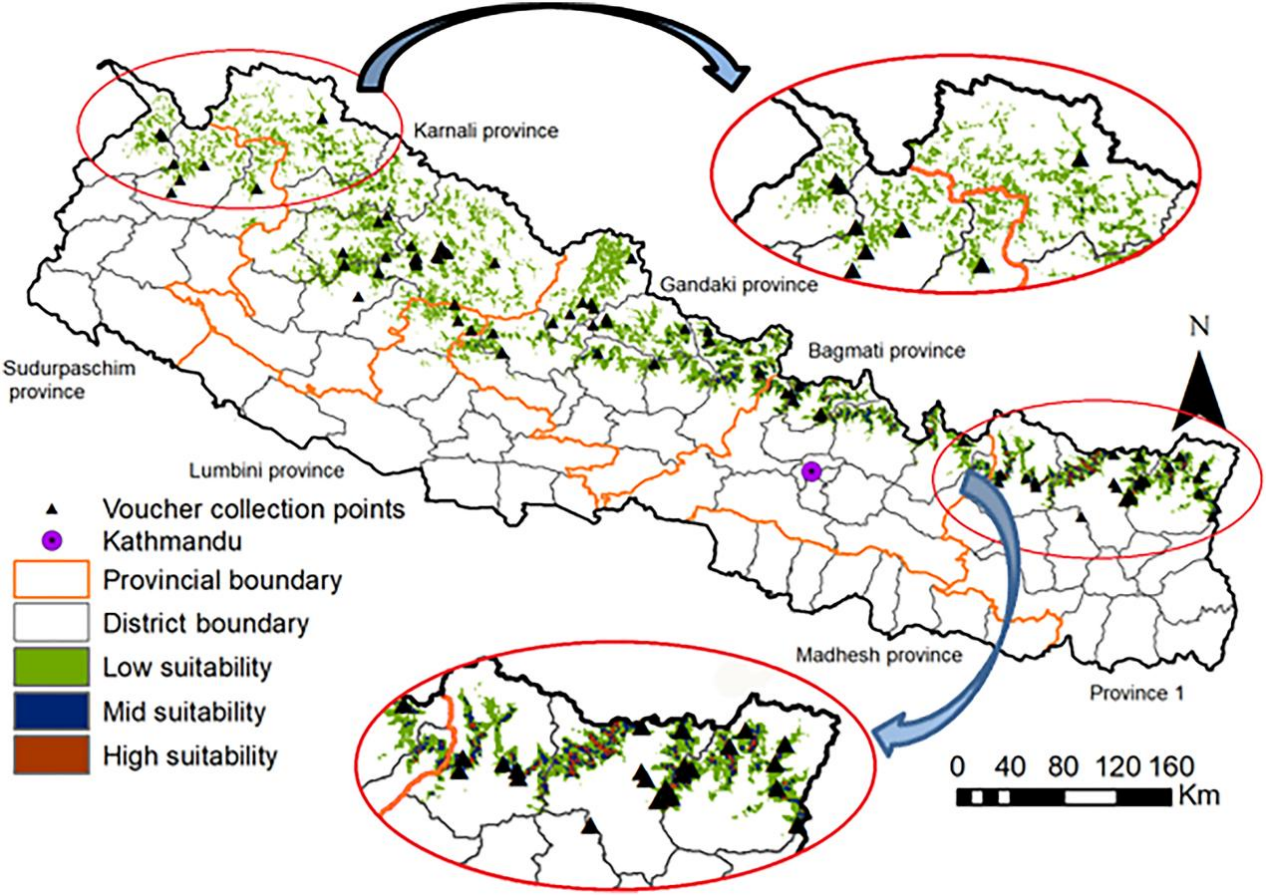
Habitat suitability for *N. jatamansi* in the Nepalese Himalaya across provinces and districts. Suitability levels are unsuitable (<25% probability of presence), low (25–50%), medium (50–75%), and high (>75%). Occurrence in 28 districts; the Karnali Province contains 33% of the suitable area and Gandaki Province around 28%.

The total area suitable for *N. jatamansi* was estimated at 16,294 km^2^ (low suitability 14,419 km^2^, medium suitability 1,608 km^2^, and high suitability 268 km^2^), equivalent to 11.0% of the country’s area (Fig. 2, Supplementary Information 4). The largest high suitability areas were in Eastern Nepal; the larger western low suitability areas are due to the increasing westward width of the Himalayan range combined with a less optimal mean temperature of the wettest quarter. Comparing the species distribution with the government district-level quota system (30) showed two differences (Supplementary Information 8). First, quotas have been assigned for three districts where the species does not occur according to our model. This indicates that some quotas are based on trader location rather than harvesting sites. Second, five districts with suitable habitat are not included in the quota system.

As inputs to Step 3 below, the adjusted suitable area was estimated at 4,609 km^2^, equivalent to 3.1% of the country’s area. The literature-derived ecological parameter estimates for the sustainability assessment were (Supplementary Information 5): an adjustment factor of 0.46 [thus estimating the actual habitat as 46% of the adjusted suitable area, reflecting the patchy distribution of the species in distinct plant communities, (31)], a conservative stock rate of 141 kg air-dry rhizomes per ha, and a sustainable yield of 10, 25, or 100% (see the three scenarios below) harvested every 5 years.

### Step 2—Trade data: production network and volumes

The production network remained similar across the 1997–1998 and 2014–2015 study years. In 2014–2015, rural households continued to collect *N. jatamansi* rhizomes exclusively in the wild on dedicated trips to remote subalpine and alpine collection areas, with an average of 106 ± 119 kg air-dried rhizomes/household/year using 13 ± 11 days (*n* = 25) from August to December. Sales to traders (*n* = 43) and domestic processors (*n* = 6) were mainly done from September through January. The rhizomes then moved to central wholesalers (*n* = 17) and additional domestic processors (*n* = 20), concentrated in the larger cities. Central wholesalers exported to regional wholesalers (*n* = 12) in India; we registered no *N. jatamansi* regional wholesalers in Tibet. The market beyond the regional wholesalers remains undocumented.

The annual national-level trade increased threefold from 1997–1998 to 2014–2015, from 377 t to 1,145 t (Table 1), equivalent to more than 1.7 billion rhizomes in 2014–2015, assuming an average air-dry rhizome weight of 0.66 g (32). The absolute amount purchased directly from harvesters by domestic processors in Nepal increased 1.8-fold, from 164 to 289 t, while the amount handled by traders quadrupled, from 212 to 856 t. Although total domestic processors' purchases increased from 201 t in 1997–1998 (22) to 354 t in 2014–2015 (28), the rising harvest is primarily export driven, increasing 4.5-fold from 176 t to 791 t (calculated as production minus the domestic industry purchase).

**Table 1.** National and province overviews of *N. jatamansi* trade in Nepal: air-dried rhizomes (t) purchased directly from harvesters by traders and domestic processors in 1997–1998 and 2014–2015, and the annual government quota.

	Trader estimate		Processor estimate		Total trade		
Province	1997–1998	2014–2015	1997–1998	2014–2015	1997–1998	2014–2015	Quota^a^
Province No. 1	57	0	33	58	90	58	26
Madhesh	0	0	0	0	0	0	0
Bagmati	90	4	22	40	112	43	8
Gandaki	4	23	45	80	49	103	46
Lumbini	3	67	4	8	8	74	43
Karnali	36	732	48	85	85	817	664
Sudurpashchim	22	30	11	19	33	49	91
Total	212	856	164	289	377	1,145	878

^a^Quota is the government's annual allowable harvest (23), excluding the quotas from Dailekh, Doti, and Pyuthan districts where the species does not occur (including these gives a total national quota of 935 t).

The increase in trade between the two observation periods occurred in western Nepal, particularly in Karnali Province supplying 22% of traded rhizomes in 1997–1998 and 71% in 2014–2015. Trade decreased in the east. District-level trade details are presented in Supplementary Information 6. Prices fluctuated around increasing average trends, indicating that demand has increased faster than supply (Supplementary Information 7). The estimated national-level trade in 2014–2015 (1,145 t, Table 1) was 30% above the government-assigned quota (878 t), with estimated trade exceeding quotas in 15 districts (Supplementary Information 8).

### Step 3—Sustainability estimates

Three sustainable harvest scenarios were identified based on two studies. A 4-year population study in north-western Nepal found higher growth rates and faster recovery in meadow populations (higher recruitment, faster vegetative growth) than in rocky-outcrop populations (slow growth, low fecundity) and recommended low harvest rates (≤25% in meadows and ≤10% in outcrops) with at least 5 years between harvests to allow population recovery (33). A 3-year regeneration study in central Nepal found that 100% rhizome harvesting (the local harvesting practice) in meadow and shrub populations followed by replanting of upper plant parts and 2 cm of the rhizome provided the fastest rhizome biomass growth and regeneration (32). This allowed us to define three scenarios for estimating sustainable harvest levels (Table 2, Supplementary Information 8): (i) The Conservative scenario: harvest of 10% of stock to reflect the vulnerability of rocky-outcrop populations to harvests, (ii) The Common scenario: harvest of 25% of stock in the less vulnerable and more common meadows, and (iii) The New harvest scenario: 100% harvest with replanting. In each scenario, there are 5 years between harvests.

**Table 2.** Estimates of sustainable harvest levels of air-dry *N. jatamansi* rhizomes (t) and shares (%) of the estimated 1997–1998 and 2014–2015 harvests in Nepal across the three sustainability scenarios.

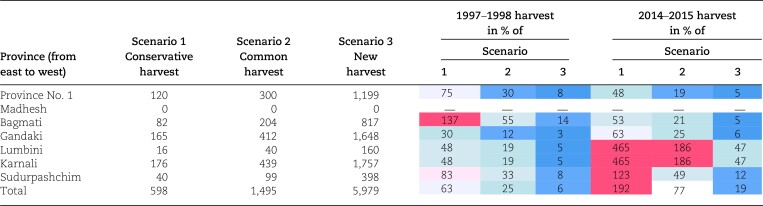

Notes: Colors illustrate the degree of sustainability: red is unsustainable (>100% of the scenario harvest level), pink is moving close to unsustainable (>80%), and blue to green is sustainable (≤80%). The two districts in Lumbini province were treated as the districts in Karnali resulting in the same shares for the two provinces.

The estimated sustainable harvest range is wide, from 598 to 1,495 t/year under existing harvesting practices to 5,979 t/year with improved practices (Table 2). As illustrated by the red color, the unsustainable harvesting pressure shifted from eastern to western Nepal between the observation years; the area subject to unsustainable harvesting increased; and the likelihood of unsustainable harvesting increased, with harvesting exceeding the sustainable levels in the Common scenario in addition to the Conservative scenario. The national-level trade in 1997–1998 was well below the sustainable harvest levels in all three scenarios; at the province level, however, harvesting in Bagmati Province (Supplementary Information 8) exceeded the Conservative scenario limit. In 2014–2015, the total national-level harvest exceeded the Conservative scenario limit by 92% while remaining below the other scenarios' limits. However, in the Karnali and Lumbini provinces, the trade in 2014–2015 exceeded the Conservative and Common scenario limits, indicating overharvesting with current collection practices. Harvesting in Sudurpashchim Province also exceeded the Conservative scenario limit. Harvesting in all districts in the Karnali and Lumbini provinces exceeded the Common scenario limit; all districts in Sudurpashchim Province exceeded the Conservative scenario limit (Supplementary Information 8). With improved practices in the new harvest scenario, none of the harvesting levels were unsustainable at any time.

To assess whether the spatial distribution modeling can be replaced with low-cost easily available proxies, we calculated the sustainability estimates using total district area (34) and district forest area (35), Supplementary Information 9. These did not provide valid estimates at a finer scale (the province or district levels); however, the total district area generated a valid national-level sustainability estimate (+0.4% and −1.1% difference for 1997–1998 and 2014–2015).

### Comparing estimated trade levels and official records

The national-level annual trade varied substantially in official government records, from 8 to 121 t (Supplementary Information 10) but was persistently far below our estimates. For example, in 2014–2015: (i) less than 7% of trade was registered (79 t out of 1,145 t) at the national level, (ii) provincial registration ranged from 0–10%, and (iii) trade was registered in only five districts (including Dailekh where the species does not occur) where the registration percentage was 5, 10, 12, and 35%. Hence, official government records cannot be used to assess trade at any geographical scale.

The CITES trade data for the period 1997–2017 (Supplementary Information 11) showed that almost all reported legal trade was wild-harvested and took place as processed products (derivatives). There was a large discrepancy between exporter and importer-reported quantities. The average total importer-reported trade was 49 kg/year, and the exporter-reported trade 73,500 kg/year with 99% from Nepal. Contrasting this, the central wholesaler export was estimated at 856,000 kg unprocessed air-dry rhizomes in 2014–2015 (Table 1) although such export is formally prohibited (36). Alternative explanations, such as stocking, appear unlikely given the low processing capacity in the country (37). Since the CITES Trade Database only captures legal trade, it cannot be used to assess actual trade levels.

## Discussion

### Wider applications of the approach

The data-driven three-component approach is structurally simple and applicable to other species. It facilitates a structured analysis of multifaceted and inherently incomplete data to assess sustainability and rationalize policy recommendations. The main challenge lies in uncovering available data, obtaining access, and then generating data where these are missing, e.g. through trade studies or inventories. Data gaps will be case specific, and closing them may require skills across different fields (ecology, economics) and can be costly; also, their relevance may not be evident to funding agencies pursuing more visible contemporary challenges, e.g. arising from climate change. However, collating existing data are cheaper and faster than generating them from scratch. In the Himalaya, there is already a solid foundation for applying the approach to other species, e.g. spatial distribution modeling has been undertaken for the widely traded *Neopicrorhiza scrophulariiflora* (38). Hence, a systematic effort should be undertaken to uncover available data for the species traded in large amounts and subject to conservation concerns (16). The total district area proxy appears valid at the national level, as a “smoke alarm” to warn of possible overharvesting. Further work could generate better low-cost proxies, e.g. district-level upland pasture area estimates for alpine species.

By combining credible district-level trade data with empirically based quantifications of species distribution, stock, and yield, we generated the currently best available estimates of the *actual* and sustainable harvest levels of *N. jatamansi*. The main challenges were a dearth of ecological parameter estimates in the literature (Supplementary Information 5); more habitat studies and inventories would make the adjustment factor and the stock estimate per unit area more robust. Also, the sustainable harvest estimates could be biased, as they are partly derived from the areas of suitable habitat: historical occurrence data may not be representative of the current species distribution, e.g. due to past overharvesting in larger regions. However, there is no evidence of such past overharvesting, including in Eastern Nepal.

### The sustainability of commercial *N. jatamansi* harvesting

Widespread early claims of unsustainability (27) appear unsubstantiated as the harvest in 1997–1998 was well below the Conservative scenario estimates for all provinces except for Bagmati (Table 2). However, the increase in annual trade between the study periods, from 377 to 1,145 t, indicates unsustainable 2014–2015 harvest levels in western Nepal, particularly in the Karnali Province. Addressing this is urgent as (i) Nepal is the main global supplier (29), (ii) the increasing prices (Supplementary Information 7) indicate rising demand, (iii) other range countries limit harvesting, such as the ban on commercial harvesting in India (17), and (iv) there are also concerns about the sustainability of harvest on the Tibetan plateau (39). Arguably, price increases, dwindling stocks, and continued wild harvesting (no cultivation) may indicate a species moving towards economic extinction (40). On the other hand, the New harvest scenario indicates a considerable potential to increase annual harvests through changed harvesting practices.

Our sustainability estimates should be considered lower-bound estimates as they derive from a double-conservative approach calculating the distribution area using the lowest probability in each suitability class and further reducing this area using an adjustment factor (reducing the potential growing area to the actual growing area). However, the traded volume estimates may also be conservative: traders have an incentive to underestimate amounts to circumvent district-level harvesting quotas and reduce tax payments. In addition, modeling suggests that the distribution area will increase with climate change in Nepal and China (41–43). We cannot quantify the net effect of these countervailing factors.

What is the best approach to promote sustainable harvesting? Species conservation requires effective regulation of who can harvest how much, when, where, and how to allow seed dispersal and regeneration, and to implement the New harvest scenario, which includes a degree of cultivating-while-harvesting. The current centralized approach of governing the harvest of *N. jatamansi*, through quotas and royalties, appears to have limited conservation impacts. Illegal trade is widespread through underreporting (in collection, transport, and export permits to evade royalty and customs payments) and the export of unprocessed rhizomes. Trade bans risk driving the trade underground rather than stopping unsustainable practices (16). Given the ease of product recognition and the fine-grained existing transport control network (forest and police check posts, customs), the failure to control illegal trade is not a technical but a systemic challenge. Rational actors are unlikely to invest in governing and improving the productivity of renewable natural resources unless they hold enforceable exclusive long-term rights (44). Since 1993, Nepal's community forestry programme has enhanced forest conservation by transferring exclusive and permanent forest management rights, plus full ownership to the resulting products, from the state to 22,415 forest user groups managing 2.4 million ha, one-third of the country's forest area (45). Decentralizing the governance of *N. jatamansi* subalpine and alpine production areas along similar lines would be an important step to improve resource governance, promote sustainable harvesting, and meet international biodiversity commitments, including under CITES and CBD's Global Biodiversity Framework to ensure that no wild species are endangered by international trade. The need for new strategies to include community-based sustainable wildlife governance has also been recognized for illegally traded wild orchids (6). However, Nepalese community forest user groups' rights are contingent on complex community forest management plans endorsed by district forest officers. Recent scrutiny of such plans found that inventory guidelines were often ignored, poorly conducted, and contained fabricated data (46). Forestry officials mainly use the legally required plans to generate informal incomes (47). Hence, representative and downwardly accountable local institutions should prepare officially recognized plans to manage wild plant populations in accordance with simple environmental standards. District-level harvesting limits can be based on our findings, while harvest limitations in individual community forests (which may include alpine meadows) can be based on already available area estimates and the sustainability parameters reported here (Supplementary Information 5). A range of complementary nonregulatory measures, such as voluntary sustainability standards and traceability approaches, can also incentivize conservation action and increase transparency (48).

The model and its application indicate that conservation policies can gain from ecological and trade data generation, integration, and upscaling. This can result in empirically informed sustainability estimates at policy relevant levels. For many species, however, investments in data generation are required before data integration and upscaling is possible. This may include establishing ecological parameter estimates, such as growing stock and yield, and collecting accurate local level trade data.

## Materials and methods

The methods below are all used to empirically assess the sustainability of trade in Nepalese *N. jatamansi* rhizomes. We modeled the species' distribution, collected existing area-based estimates of stock and yield rates, generalized trade data for two observation years (1997–1998 and 2014–2015), and combined these to assess the ecological sustainability at district, province, and national levels.

### Data collection: step 1, ecological data

We undertook habitat niche modeling using 89 unique presence records from Tribhuvan University Central Herbarium, the National Botanical Garden, Tokyo University, Royal Botanical Garden Edinburgh, Muséum National d'Histoire Naturelle in Paris, Royal Botanic Gardens Kew, and the British Museum of Natural History. Removing duplicate points to avoid spatial autocorrelation and bias caused by oversampled regions, using a minimum distance of 10 km between points (42), yielded 39 points used to calibrate the model and identify relationships between species occurrence and environmental variables. Bioclimatic variables were obtained from a global data set (49), and topographic variables from a Digital Elevation Model based on the Shuttle Radar Topographic Mission (Supplementary Information 2). The final dataset had a spatial resolution of 30 arc seconds. As collinearity may be high among variables in small geographical areas, we calculated correlation using ENM Tools v1.4.4 (50) and included six bioclimatic and three topographical variables with a Pearson correlation coefficient <0.8 (Supplementary Information 2).

The potential distribution based on patchy presence-only data is best predicted using the Maximum Entropy approach (51). We used 75% of the presence data for calibration and 25% for model validation, with a single regularization multiplier and 10,000 randomly distributed background points. The model was calibrated using bootstrapping with 50 replications, a convergence threshold of 0.00001, and up to 5,000 iterations. We used the MaxEnt algorithm’s linear, quadratic, product threshold, and hinge features. Model performance was evaluated using the area under curve (AUC) receiver operating characteristic giving a single measure of overall accuracy independent of any threshold (52). MaxEnt modeling successfully mapped the potential distribution area: training AUC value of 0.942 and test AUC value of 0.885 (53). We assessed which variables significantly affected the probability of presence using response curves (51), Supplementary Information 3. The output distribution rasters were classified into four classes: unsuitable (<25% probability of presence), low (25–50%), medium (50–75%), and high suitability (>75%). This represents suitability and not the actual occupied area. All analysis was done using ArcGIS 10.5. We calculated a conservative adjusted suitability area at the district level as the sum of probabilities multiplied by areas in the three suitability classes using the low-end probabilities (e.g. 25% of the area of low suitability); we used this conservative estimate (on average 28% of total suitable habitat area) to generalize trade data and assess sustainability.

An overview of parameter estimates from the literature used for adjusting from potential to actual distribution area, growing stock, annual sustainable yield, and rotation period is presented in Supplementary Information 5. This resulted in an application of an adjustment factor of 0.46, a conservative low stocking rate of 141 kg air-dry rhizomes per ha, and a sustainable yield of 10, 25, or 100% of the stocking (see scenarios below) harvested every 5 years.

### Data collection: step 2, trade data

Trade data were collected from June 2015 to September 2016 in 15 randomly selected case districts, one in each of the 15 cells formed by the country’s five ecological and three physiographic zones (Supplementary Information 12) for the case year 2014–2015 (the Nepalese year 2071). This repeated a similar survey for 1997–1998 undertaken in 1999 (18, 47). Hence, the trade data required to estimate trade levels are only available for 1997–1998 and 2014–2015. In each case district, all local traders were identified, tracked, and interviewed (*n* = 166 and *n* = 382 in 1997–1998 and 2014–2015) using structured questionnaires. Harvesters (*n* = 639 and *n* = 540) encountered en route were interviewed. All central wholesalers in Nepal were interviewed (*n* = 90 and *n* = 58 in the two periods), as were regional wholesalers in India (*n* = 53 and 30; in Calcutta, Siliguri, Lucknow, Gorakhpur, Kanpur, Tanakpur, Haridwar, and Delhi). In 2014–2015, the study also included regional wholesalers in Tibet (*n* = 40; in Puran, Gyilong, Shigatse, and Lhasa). In 1997–1998, the entire population of 16 domestic processors was interviewed; in 2014–2015, from a total population of 233 active processors in Nepal (54), 79 were interviewed to estimate purchases directly from harvesters. Hence, there is no double counting: the total amount traded is the sum of collector direct sales to traders, central wholesalers, and processors (thus excluding sales from traders and central wholesalers to processors). Also, data registration is only of direct sales from harvesters, of material harvested in the case year [(55), including specification of harvest month], eliminating problems of recording stocks (a minor problem; we are not aware of any examples of harvesters stocking *N. jatamansi* rhizomes between years).

We also obtained the government-approved annual allowable harvest per district (30), the district-level data on officially registered legal trade from 2008/09 to 2015/16 (Supplementary Information 10, data not available for other years), and the annual data on international trade from the CITES Trade Database from 1997–2017 (Supplementary Information 11). Lastly, to understand price development over time, we obtained central (Nepalgunj and Kathmandu) and regional (Delhi, Kolkata, Lucknow, and Tanakpur) wholesaler price data from 2002 to 2020 from a range of sources (Supplementary Information 7).

### Data analysis: Step 3

The ecologically sustainable harvest estimates were calculated at the district level:


$$Q_{i}=a_{i}A_{i}S_{i}Y_{j}R_{k}$$ (1)

Where Q_i_ is the sustainable harvest of air-dry rhizomes (kg) in district *i* (*i* = 1, 2,…,77); *a_i_* is the adjusted suitable growing area (ha) in district *i*; *A_i_* is the adjustment factor reducing the suitable area to the actual estimated growing area in district *i*; *S_i_* is the growing stock per hectare (kg) in district *i*; *Y_j_* is the amount (%) of the growing stock that can be harvested in scenario *j* (*j* = 1, 2, 3); *R_k_* is the rotation factor for the *k* years between harvesting the same population (calculated as 1/*k*, e.g. 0.2 for a 5-year rotation period). Vegetation-level analyses (31, 56) indicate most occurrences outside rocky outcrops. Using a rotation period of 5 years, Scenario 1 (Conservative) allows a 10% harvest of stock to protect the more sensitive rocky-outcrop populations (33), Scenario 2 (Common) 25% harvest allowing sustainable harvest in meadows (33), and Scenario 3 (New harvest) 100% harvesting with the replanting of upper plant parts and 2 cm of the rhizome, shown to provide the fastest regeneration and rhizome biomass growth (32).

For each case year (1997/1998 and 2014/2015), the 15 district-level trade data were generalized to the national level (62 additional districts) using the recorded volumes in the case districts and the ratio of suitable growing areas between case and noncase districts (Supplementary Information 6). Specifically:


$$Q_{jy}=(a_{j}/a_{c})Q_{cy}$$ (2)

Where *Q_jy_* is the estimated amount (kg) of air-dry rhizomes traded from noncase district *j* (*j* = 1, 2,…,62) in study year *y* (1997/1998 or 2014/2015); *a_j_* is the adjusted suitable growing area (ha) in noncase district *j*; *a_c_* is the adjusted suitable growing area in case district *c* (*c* = 1, 2,…,15) in the cell containing district *j* or the nearest neighbor; and *Q_cy_* is the reported amount (kg) of air-dry rhizomes purchased from harvesters (by traders, central wholesalers, and processors) in case district *c* in study year *y*. As processors could not specify the geographical origin of their supplies, the national amount was distributed to each district using its share of the total suitable growing area. Access to the 1997–1998 dataset (29) allowed recalculations to enable direct comparisons between the two case years. To investigate if low-cost readily available proxies for the suitable growing area could be used, we substituted that data with district-level (i) total area (34) and (ii) total forest area (35). Furthermore, we compared our trade estimates with the officially registered trade for each district, and our export estimates with international trade reported to CITES. All prices were adjusted for inflation, using the Consumer Price Index for Nepal, to the base year 2014–2015.

## Supplementary Material

pgad328_Supplementary_Data

## Data Availability

The data underlying this article are publically available in the Electronic Research Data Archive (ERDA) at the University of Copenhagen. The digital object identifier is https://doi.org/10.17894/ucph.48227096-1bcf-4c57-a7c5-d478bcef59bd.

## References

[1] Brondizio ES , Settele J, Díaz S, Ngo HT. 2019. Global assessment report on biodiversity and ecosystem services of the intergovernmental science-policy platform on biodiversity and ecosystem services. Bonn: IPBES.

[2] Fromentin JM , Fromentin JM, Emery MR, Donaldson J, Danner MC. 2022. Chapter 1: Setting the scene. In: Fromentin JM. Emery MR. Donaldson J. Danner MC. Hallosserie A. Kieling D, et al, editors. Thematic assessment report on the sustainable use of wild species of the intergovernmental science-policy platform on biodiversity and ecosystem services. Bonn: IPBES Secretariat. p. 4–55.

[3] Maxwell SI , Fuller RA, Brooks TM, Watson JEM. 2016. Biodiversity: the ravages of guns, nets and bulldozers. Nature. 536:143–145. 27510207 10.1038/536143a

[4] Joppa LN , et al 2016. Filling in biodiversity threat gaps. Science. 352(6284):416–418. 27102469 10.1126/science.aaf3565

[5] Ingram DJ , et al 2018. Assessing Africa-wide pangolin exploitation by scaling local data. Conserv Lett. 11(2):1–9.

[6] Ticktin T , et al 2020. Synthesis of wild orchid trade and demography provides new insight on conservation strategies. Conserv Lett. 13:e1269.

[7] Leão TCC , Lobo D, Scotson L. 2017. Economic and biological conditions influence the sustainability of harvest of wild animals and plants in developing countries. Ecol Econ. 140:14–21.

[8] Margulies JD , et al 2019. Illegal wildlife trade and the persistence of “plant blindness”. Plants, People, Planet. 1(3):173–182.

[9] Meilby H , et al 2014. Are forest incomes sustainable? Firewood and timber extraction and forest productivity in community managed forests in Nepal. World Dev. 64:S113–S124.

[10] Cunningham AB , Brinckmann JA, Yang X, He J. 2019. Introduction to the special issue: saving plants, saving lives: trade, sustainable harvest and conservation of traditional medicinals in Asia. J Ethnopharmacol. 229:288–292. 30326261 10.1016/j.jep.2018.10.006

[11] Hinsley A , et al 2020. Building sustainability into the Belt and Road initiative’s traditional Chinese medicine trade. Nat Sustain. 3:96–100.

[12] Christenhusz MJM , Byng JW. 2016. The number of known plant species in the world and its annual increase. Phytotaxa. 261(3):201–217.

[13] von Wettberg E , Davis TM, Smýkal P. 2020. Editorial: Wild plants as source of new crops. Front Plant Sci. 11:591554. 33014007 10.3389/fpls.2020.591554 PMC7516029

[14] CITES, The CITES species . Convention on international trade in endangered species of wild Fauna and Flora, https://cites.org/eng/disc/species.php [accessed 2023 Jan 16].

[15] Smith-Hall C , et al 2020. Trade and conservation of medicinal and aromatic plants—an annotated bibliography for Nepal. Kathmandu: Sopan Press.

[16] Pyakurel D , Smith-Hall C, Bhattarai-Sharma I, Ghimire SK. 2019. Trade and conservation of Nepalese medicinal and aromatic plants, fungi, and lichen. Econ Bot. 73:505–521.

[17] Chauhan HK , Oli S, Bisht AK, Meredith C, Leaman D. 2021. Review of the biology, uses and conservation of the critically endangered endemic Himalayan species *Nardostachys jatamansi* (Caprifoliaceae). Biodivers Conserv. 30:3315–3333.

[18] Dhiman N , Bhattacharya A. 2020. *Nardostachys jatamansi* (D. Don) DC.—challenges and opportunities of harnessing the untapped medicinal plant from the Himalayas. J Ethnopharmacol. 246:112211. 31533076 10.1016/j.jep.2019.112211

[19] Kaur H , et al 2020. *Nardostachys jatamansi* (D. Don) DC.—an invaluable and constantly dwindling resource of the Himalayas. South Afr J Bot. 135:1–16.

[20] Rehman T , Ahmad S. 2019. *Nardostachys chinensis* Batalin: a review of traditional uses, phytochemistry, and pharmacology. Phytother Res. 33:2622–2648. 31359527 10.1002/ptr.6447

[21] Olsen CS , Larsen HO. 2003. Alpine medicinal plant trade and Himalayan mountain livelihood strategies. Geogr J. 169(3):243–254.

[22] Olsen CS , Helles F. 2009. Market efficiency and benefit distribution in medicinal plant markets: empirical evidence from South Asia. Int J Biodivers Sci Manag. 5(2):53–62.

[23] Pyakurel D , Bhattarai Sharma I, Smith-Hall C. 2018. Patterns of change: the dynamics of medicinal plant trade in far-western Nepal. J Ethnopharmacol. 224:323–334. 29885362 10.1016/j.jep.2018.06.004

[24] Aryal KR , et al 2023. Contribution of medicinal and aromatic plants on gross domestic product in Karnali Province, Nepal. J Resour Ecol. 14(5):1104–1112.

[25] Chapagain A , Wang J, Pyakurel D. 2021. An overview of Nepalese medicinal plant trade with China. Int J Environ Sci Nat Resour. 28(1):556228.

[26] Lamichhane R , Gautam D, Miya MS, Chhetri HB, Timilsina S. 2021. Role of non-timber forest products in national economy: a case of Jajarkot District, Nepal. Grassroots J Nat Resour. 4(1):93–105.

[27] Larsen HO , Olsen CS. 2007. Unsustainable collection and unfair trade? Uncovering and assessing assumptions regarding central Himalayan medicinal plant conservation. Biodivers Conserv. 16:1679–1697.

[28] Dalby A . 2000. Dangerous tastes: the story of spices. London: British Museum Press.

[29] Olsen CS . 2005. Trade and conservation of Himalayan medicinal plants: *Nardostachys grandiflora* DC and *Neopicrorhiza scrophulariiflora* (Pennell) Hong. Biol Conserv. 125:505–514.

[30] DFSC . National quota fixation for Jatamansi (*Nardostachys jatamansi* DC) ensuring sustainable management and conservation in Nepal. Department of Forests and Soil Conservation. Kathmandu (undated).

[31] Smith-Hall C . 2020. Creating a historical baseline: economically important alpine plant communities in central Himalaya, Nepal. Botanica Orientalis. 14:1–13.

[32] Larsen HO . 2005. Impact of replanting on regeneration of the medicinal plant *Nardostachys grandiflora* DC. (Valerianaceae). Econ Bot. 59(3):213–220.

[33] Ghimire SK , Gimenez O, Pradel R, McKey D, Aumeeruddy-Thomas Y. 2008. Demographic variation and population variability in a threatened Himalayan medicinal and aromatic herb *Nardostachys grandiflora*: matrix modelling of harvesting effects in two contrasting habitats. J Appl Ecol. 45(1):41–51.

[34] CBS . 2020. Statistical year book of Nepal 2019. Kathmandu: Central Bureau of Statistics.

[35] DFRS . 2018. Forest cover maps of local levels (753) of Nepal. Kathmandu: Department of Forest Research and Survey.

[36] MFSC . 1995. Forest regulation. Kathmandu: Ministry of Forests and Soil Conservation.

[37] Caporale F , Mateo-Martín J, Usman F, Smith-Hall C. 2020. Plant-based sustainable development—the expansion and anatomy of the medicinal plant secondary processing sector in Nepal. Sustainability. 12(14):5575.

[38] Poudeyal M , et al 2021. Does resource availability coincide with exploitation patterns? Inference from distribution and trade of *Neopicrorhiza scrophulariiflora* (Pennell) D.Y. Hong in the Nepalese Himalayas. J Appl Res Med Aromat Plants. 22:100292.

[39] Zhao J , et al 2023. Does higher demand for medicinal plants lead to more harvest? Evidence from the dual trade of *Nardostachy jatamansi* and *Fritillaria cirrhosa* and Tibetan people's harvesting behaviour. Front Ecol Evol. 11:1145928.

[40] Madsen ST , Smith-Hall C. 2023. Wild harvesting or cultivation of commercial environmental products: a theoretical model and its application to medicinal plants. Ecol Econ. 205:107701.

[41] Li J , et al 2019. Simulating the effects of climate change across the geographical distribution of two medicinal plants in the genus Nardostachys. PeerJ. 7:e6730. 31024763 10.7717/peerj.6730 PMC6474333

[42] Rana SK , et al 2020. Climate-change threats to distribution, habitats, sustainability and conservation of highly traded medicinal and aromatic plants in Nepal. Ecol Indic. 115:106435.

[43] Kunwar RM , et al 2023. Distribution of important medicinal plant species in Nepal under past, present, and future climatic conditions. Ecol Indic. 146:109879.

[44] Ostrom E . 2009. A general framework for analysing sustainability of social-ecological systems. Science. 325(5939):419–422. 19628857 10.1126/science.1172133

[45] MoFE . 2021. Current status of community based forest management (CBFM) in Nepal 2020. Kathmandu: Ministry of Forests and Environment.

[46] Baral S , et al 2018. Politics of getting the numbers right: community forest inventory of Nepal. For Policy Econ. 91:19–26.

[47] Basnyat B , Treue T, Pokharel RK, Lamsal LN, Rayamajhi S. 2018. Legal-sounding bureaucratic re-centralisation of community forestry in Nepal. For Policy Econ. 91:5–18.

[48] Timoshyna A , Furnell S, Harter D. 2019. CITES and voluntary certification for wild medicinal and aromatic plants. TRAFFIC Bulletin. 31(2):79–88.

[49] Fick SE , Hijmans RJ. 2017. Worldclim 2: new 1 km spatial resolution climate surfaces for global land areas. Int J Climatol. 37(12):4302–4315.

[50] Warren DL , Glor RE, Turelli M. 2010. ENMTools: a toolbox for comparative studies of environmental niche models. Ecography. 33:607–611.

[51] Phillips SJ , Dudík M. 2008. Modeling of species distributions with MaxEnt: new extensions and a comprehensive evaluation. Ecography. 31:161–175.

[52] Deleo JM , Ayyub BM. 1993. Receiver operating characteristic laboratory (ROCLAB): software for developing decision strategies that account for uncertainty. In: Ayyub BM, editors. Proceedings of the second international symposium on Uncertainty Modeling and Analysis. Los Alamitos (CA): Computer Society Press. p. 318–325.

[53] Franklin J . 2010. Mapping species distributions. Cambridge: Cambridge University Press.

[54] Chapagain A , et al 2019. A population list of medicinal plant processing enterprises in Nepal. IFRO documentation series 2019/3. Copenhagen: University of Copenhagen.

[55] Smith-Hall C , et al 2018. Data collection instruments and procedures for investigating national-level trade in medicinal and aromatic plants. IFRO documentation 2018/2. Copenhagen: University of Copenhagen.

[56] Ohba H , Iokawa Y, Sharma LR, editors. 2008. Flora of Mustang, Nepal. Tokyo: Kodansha Scientific Ltd.

