# Supplementary material for: The sustainability of trade in wild plants—A data-integration approach tested on critically endangered Nardostachys jatamansi
Source: PNAS Nexus. 2023 Nov 7;2(11):pgad328. doi: 10.1093/pnasnexus/pgad328 (PMC10635652; doi:10.1093/pnasnexus/pgad328)
Supplement: pgad328_Supplementary_Data [file pgad328_supplementary_data.docx]

**Supporting Information for**

The sustainability of trade in wild plants – a data-integration approach tested on critically endangered *Nardostachys jatamansi*

Carsten Smith-Hall^1,*^, Dipesh Pyakurel^2^, Henrik Meilby^1^, Mariève Pouliot^1^, Puspa L. Ghimire^3^, Suresh Ghimire^4^, Sofia T. Madsen^5^, Yagya R. Paneru^6^, Bhishma P. Subedi^3^, Anastasiya Timoshyna^7^, and Thorsten Treue^1^

^1^ Department of Food and Resource Economics, Faculty of Science, University of Copenhagen, 1958 Frederiksberg, Denmark
^2^ Resources Himalaya Foundation, Lalitpur, Nepal
^3^ Asia Network for Sustainable Agriculture and Bioresources, Kathmandu, Nepal
^4^ Central Department of Botany, Tribhuvan University, Kirtipur, Nepal
^5^ Department of Planning, University of Aalborg, Copenhagen, DK
^6^ National Herbarium and Plant Laboratories, Lalitpur, Nepal
^7^ TRAFFIC International, Cambridge, UK

*Carsten Smith-Hall, Rolighedsvej 23, 1958 Frb C, +45 21308683

**Email:**  cso@ifro.ku.dk

**This PDF file includes:**

Supporting text

Figures S1 to S12

Tables S2 to S12

References

Table of Contents

[**Supplementary Information 1**. The case species *Nardostachys jatamansi* (D. Don) DC. 3](#_Toc147467123)

[**Supplementary Information 2.** Overview of bioclimatic variables used in spatial distribution modelling for *N. jatamansi* in Nepal 7](#_Toc147467124)

[**Supplementary Information 3.** Training gains, response curves, and relative contribution of environmental factors in modelling the probability of the presence of *N. jatamansi* in the Nepalese Himalayas 9](#_Toc147467125)

[**Supplementary Information 4.** Estimated area of habitats suitable for *N. jatamansi* per province and district in the Nepalese Himalayas 13](#_Toc147467126)

[**Supplementary Information 5.** Overview of ecological parameter estimates for sustainability assessment of *N. jatamansi* 14](#_Toc147467127)

[**Supplementary Information 6**. Province and district-level estimates of *N. jatamansi* trade in Nepal (kg air-dried rhizomes) in 1997-98 and 2014-15 16](#_Toc147467128)

[**Supplementary Information 7**. Overview of price developments for air-dried *N. jatamansi* rhizomes in Nepal and India 18](#_Toc147467129)

[**Supplementary Information 8**. District, province, and national level sustainability estimates for the trade in air-dried rhizomes of *N. jatamansi* in 1997-98 and 2014-15, Nepal (kg) 22](#_Toc147467130)

[**Supplementary Information 9.** National, provincial, and district level comparisons of *N. jatamansi* trade volumes (kg): the adjusted suitability approach results vs using the two low-cost proxies: total district area and district forest area 24](#_Toc147467131)

[**Supplementary Information 10**. District, province, and national level trade in the air-dried rhizomes of *N. jatamansi* from fiscal years 2008/09 to 2015/16, Nepal (kg), according to Department of Forests records (DoF 2009 to 2016) 28](#_Toc147467132)

[**Supplementary Information 11**. CITES Trade Data analysis for *N. jatamansi* for 1997-2017 29](#_Toc147467133)

[**Supplementary Information 12.** Fieldwork districts, physiographic zones, and development regions 32](#_Toc147467134)

[**References** 35](#_Toc147467135)

**Supplementary Information 1**. The case species *Nardostachys jatamansi* (D. Don) DC.

*Nardostachys jatamansi* (D. Don) DC. (syn. *N. grandiflora* DC., *N. grandiflora*Wall. ex DC*.,* *N. gracilis* Kitam, *N. chinensis* Batalin, *Fedia grandiflora* Wall., *Patrinia jatamansi* D. Don, *Valeriana jatamansi* auct. non Jones, *V. jatamansi* D. Don; 1-3) belongs to the family Caprifoliaceae (previously Valerianaceae). It is an aromatic herbaceous plant 10-60 cm tall. The rhizomes are short, dark grey, woody, thickened, and covered by reddish-brown fibrous stalks of withered leaves, about 0.5 to 1 cm thick and 10 to 15 cm long. The leaves arise in clusters almost from the ground surface in rosette form. Inflorescences are borne on terminal capitulum. Flowers are often light purple and campanulate. The growth season runs from May to late October. Flowering occurs in June-August and fruiting in August-November (4).

The rhizomes are used in many products, including in Ayurveda, Traditional Chinese Medicine, and popular products such as incense and hair tonics (5, 6). While many uses are known, there are no consumer surveys that allow an assessment of the most important end products and, thus, an informed discussion of likely future demand. However, given the species integration into traditional medicine systems that are increasingly popular (7-9), it appears likely that demand will increase in the short and middle terms. Recent reviews provide updated detailed ethnobotanical and pharmacological knowledge for the species (6, 10, 11).

All three variants of *N. jatamansi* (12) are reported from Nepal (Fig. S1), where the species is distributed throughout the country's alpine and subalpine regions in meadows, thickets, and on rocky slopes with coarse sandy loam soil (3, 4). *N. jatamansi* has also been reported from India, Bhutan, China, and upper Burma (13) from 3200–5000 masl (14) but may also occur in Afghanistan and Pakistan (5, 15). The common trade name is jatamansi; other localised folk names in Nepal are balchad, bhutle, bhultya (Nepali, Jumli), poi (Tamang), pangbo, pangbin (Limbu), pangpo (Sherpa), and pangpoi (Tibetan) (1, 16).

Conservation: there are no studies tracking *N. jatamansi* populations over time, but they are argued to be declining due to overharvesting and habitat loss (1), the species growth pattern, morphology, and reproductive biology making it sensitive to harvesting (17), putting both species and livelihoods at risk. Secondary threats include habitat fragmentation and degradation, overgrazing, and forest fires (18). Overharvesting has been claimed as the major cause of resource depletion (19).

*N. jatamansi* was added to the Convention on International Trade in Endangered Species (CITES) Appendix II (exports must thus be sustainably harvested) in 1997. It is a critically endangered species as per the IUCN Red List (20). In response to the perceived wild population decline due to overexploitation, Nepal banned the export in 1995 via publication of a notification in the Nepal Gazette (under rules 12 and 13(2) of the forest regulation, 1995) on 3^rd^ April 1995. Later, in 2001, it was amended, and export was allowed in the processed form (clause 2 of the Nepal Gazette vol 3, section 51, No. 36) (1). This species is officially identified as an economic development priority (21), and the Nepal government has the declared goal to pursue medicinal plant-based development (22). Recently, *N. jatamansi* has been identified as Nepal's most vulnerable traded medicinal plant because of premature harvesting and overharvesting – unsustainable harvest happens through the extraction of all individuals in a specific location before seed dispersal hampering sexual reproduction and regeneration from rhizomes (14).

Production and trade: there are several old reports of trade of *N. jatamansi* from Nepal (e.g. 23). Many recent studies mention the export from Nepal, the volume ranging from 100 tons (18), 100 – 500 tons (24), and 500 – 1000 (25). In 2018, it was estimated that 935.3 metric tons of jatamansi can be annually harvested from Nepal (26), with issued collection permits amounting to 305 tons in the fiscal year 2020/21 (unpublished data from the CITES Management Authority, DFSC on 6^th^ December 2021). Taplejung, Sankhuwasabha, Solukhumbu, Dolakha, Rasuwa, Dhading, Gorkha, Mustang, Jajarkot, Dolpa, Mugu, Jumla, and Humla have been reported as the major production districts (18, 19, 27-29). The Department of Forests (DoF) data from 2008/09 to 2015/16 showed jatamansi collection in 18 districts with Jumla, Humla, Dolpa, Mugu, Jajarkot, Bajhang, Bajura, Rukum, Gorkha, Lamjung, and Dhading having the highest annual trade quantity (30), see also SI 10. Other studies have reported district-level trade quantities, e.g., 30 tons from the eastern hills (via Hile and Basantapur) in FY 1991/92 (31), 25.3 – 83.6 tons from Gorkha in FY 1994/95 (32), 2.7 tons from Rasuwa in 2005/06 (33), 7.0 and 6.3 tons from Darchula districts in FY 1997/98 and FY 2014/15 respectively (9).

In the mid-1990es, it was estimated that 80 tons of *N. jatamansi* rhizomes were processed annually in Nepal (34). The processing quantity significantly increased after the ban on the export of unprocessed rhizomes, with an estimate (35) that 75% of the total production was domestically processed. A recent study estimated that 354 tons of dried rhizomes were purchased by processors in Nepal (36). Jatamansi is mainly collected from Karnali province (DFSC undated) and primarily processed in Nepalgunj (in Banke District) and Krishnanagar (Kapilbastu District). The rhizomes contain essential (spikenard) oil making up from 0.57 – 1.63% of the dry rhizome weight, varying with geographical location and habitat, with oil content reaching 2% under carefully managed conditions (18, 28). Spikenard oil is exported to India, Japan, Switzerland, and the USA, and the marc (processed rhizomes) is exported to India, Pakistan, Bangladesh, and UAE (37).

Production network: the trade in *N. jatamansi* from Nepal takes place throughout the high mountains, with products entering the same global production network. It is estimated that over 15,000 households, often poorer, harvest jatamansi, contributing to 20–25% of their average annual income (38). Harvesters sell the air-dried rhizomes to sub-local traders who operate from villages or occasionally visit collection sites. Harvesters also sell to local traders (operating from district headquarter, major market centres, or road heads) or essential oil processors. In all cases, harvesters often receive an advance payment in cash or kind. The sub-local traders sell to local traders or processors. Local traders obtain the legally required collection permit from the CITES unit, Department of Forests and Soil Conservation and subsequently a transport permit from the division forest offices. Jatamansi from Humla is air-lifted to Surkhet or Nepalgunj and transported to Nepalgunj via the road network from most other districts. Jatamansi collected from the western part of Nepal is processed chiefly at Nepalgunj to produce spikenard oil. Processors do not receive payments in advance. Processors at Nepalgunj export the oil to India, followed by European countries. The marc (residue after extracting the essential oil) is exported to India, Pakistan, and Bangladesh to prepare incense sticks.

Division Forest Offices (provincial government) issues collection and export permits for non-CITES-listed plants but the collection permit for CITES-listed plants is issued by the Department of Forests and Soil Conservation (federal level), which, in turn, receives the royalty, creating a conflict between federal and district units regarding resource stewardship and benefit-sharing. The jatamansi production network in Nepal may be dominated by processors with access to export spikenard oil prices (not available to sub-local and local traders).

Illegality issues: the government of Nepal issues collection permits from mid of September to mid of November, but harvesting starts in August. Collection permits are obtained by local traders, not harvesters who remain ignorant of the issued quota. The trade of surplus quantity (collected beyond the allocated quota) is facilitated by local traders making unofficial payments to officials of the Division Forest Office. Local traders then receive the transport permit to transport products out of the district of origin. Rent-seeking is also exercised during the weighing of the products and at check posts during transportation.

| 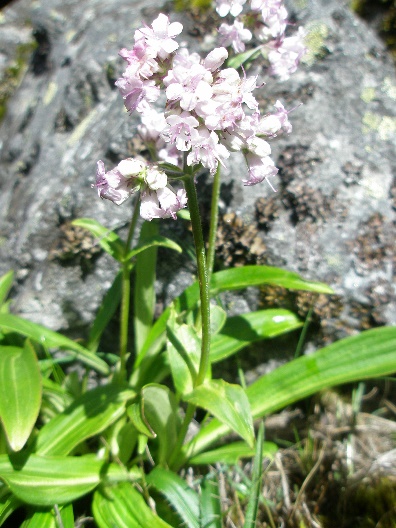 | 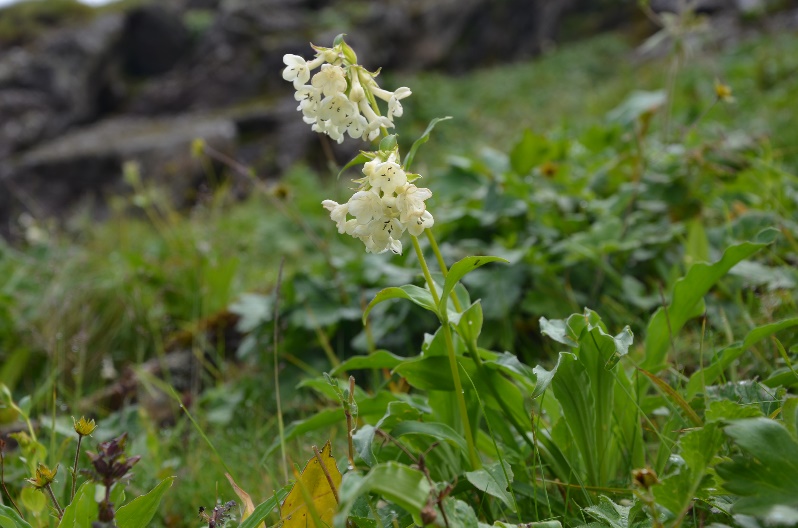 | 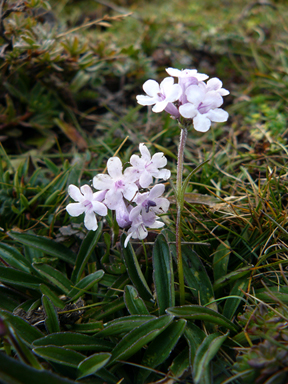 |
| --- | --- | --- |
| (a) jatamansi type | (b) grandiflora type | (c) gracilis type |

Fig. S1. The three variants of *N. jatamansi*: (a) jatamansi in moist meadows, (b) grandiflora in rocky areas, and (c) gracilis in dry meadows. Photo credits: Dipesh Pyakurel (a, b) and Suresh K. Ghimire (c).

**Supplementary Information 2.** Overview of bioclimatic variables used in spatial distribution modelling for *N. jatamansi* in Nepal

Table S2a. The 19 bioclimatic variables used as the starting point for predicting the distribution of *N. jatamansi* in the Nepalese Himalayas

| bio1 | Annual mean temperature |
| --- | --- |
| bio2^٭^ | Mean diurnal range (Mean of monthly (max. temp. – min. temp.)) |
| bio3^٭^ | Isothermality (bio2/bio7) (* 100) |
| bio4^٭^ | Temperature seasonality (standard deviation *100) |
| bio5^٭^ | Maximum temperature of the warmest month |
| bio6^٭^ | Minimum temperature of the coldest month |
| bio7 | Temperature annual range (bio5 - bio6) |
| bio8 | Mean temperature of wettest quarter |
| bio9 | Mean temperature of driest quarter |
| bio10 | Mean temperature of warmest quarter |
| bio11 | Mean temperature of coldest quarter |
| bio12 | Annual precipitation |
| bio13 | Precipitation of wettest month |
| bio14 | Precipitation of driest month |
| bio15 | Precipitation seasonality (Coefficient of variation) |
| bio16 | Precipitation of wettest quarter |
| bio17 | Precipitation of driest quarter |
| bio18 | Precipitation of warmest quarter |
| bio19 | Precipitation of coldest quarter |

^٭^Derived from maximum and minimum temperatures

Table S2b. The nine predictor variables used to calibrate the *N. jatamansi* distribution model for Nepal

| Category | Variable description |
| --- | --- |
| *Climatic* | Source: Worldclim |
| Bio 3 | Isothermality (Bio 2^1^/Bio 7) × 100 |
| Bio 7 | Temperature annual range (Bio 5-Bio 6) |
| Bio 8 | Mean temperature of the wettest quarter |
| Bio 15 | Precipitation seasonality (coefficient of variation) |
| Bio 17 | Precipitation of driest quarter |
| Bio 18 | Precipitation of warmest quarter |
| *Topographic* | Source: DEM SRTM |
| Aspect | Aspect in degrees (orientation of slope) |
| Elev. | Elevation (in meters) |
| Slope | Slope in degrees (incline of the surface) |

Units: temperature in degree Celsius, precipitation in mm
^1^ Bio 2 is the mean diurnal range (mean of monthly (max temp - min temp)). Bio 5 is the max temperature of the warmest month, and Bio 6 is the min temperature of the coldest month

**Supplementary Information 3.** Training gains, response curves, and relative contribution of environmental factors in modelling the probability of the presence of *N. jatamansi* in the Nepalese Himalayas


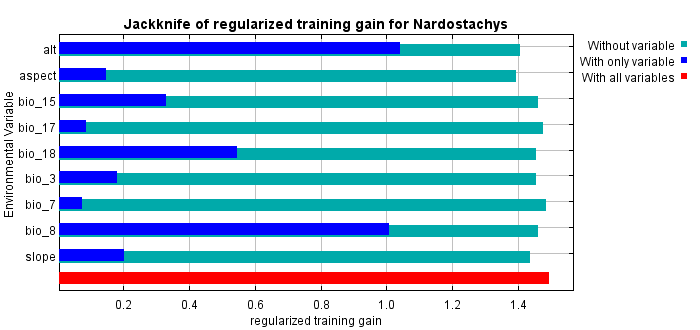


Fig. S3a. Jackknife test to estimate the relative influence of individual predictors (variables) on the distribution of *N. jatamansi* in the Nepalese Himalayas**.** The variables are presented in Table S2a.

| 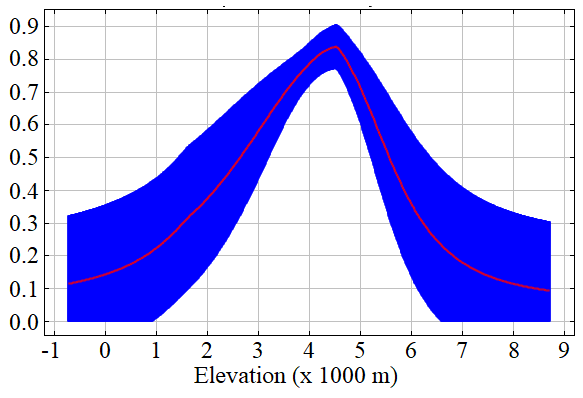 | 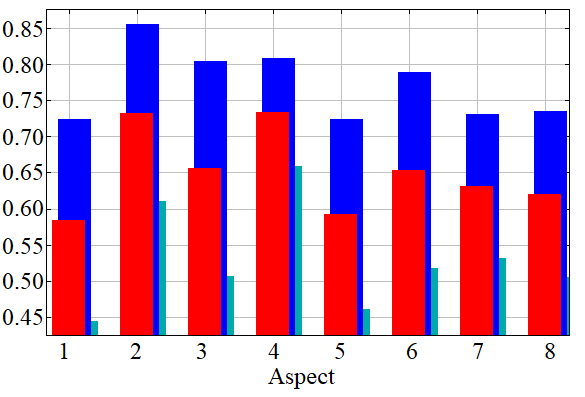 |
| --- | --- |
| 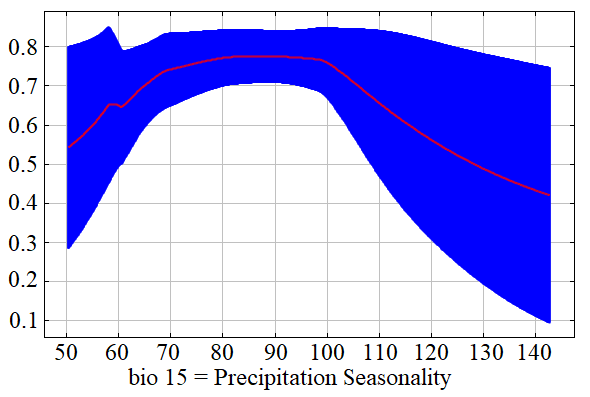 | 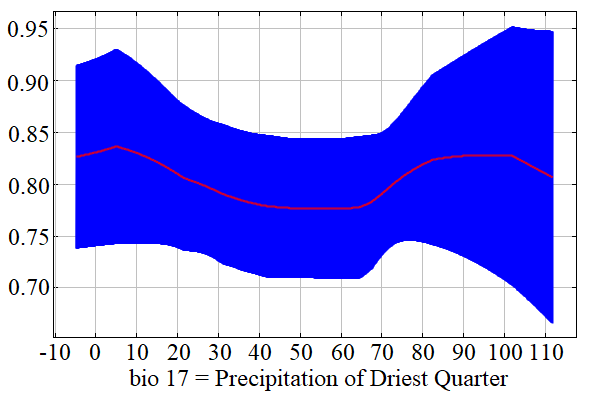 |
| 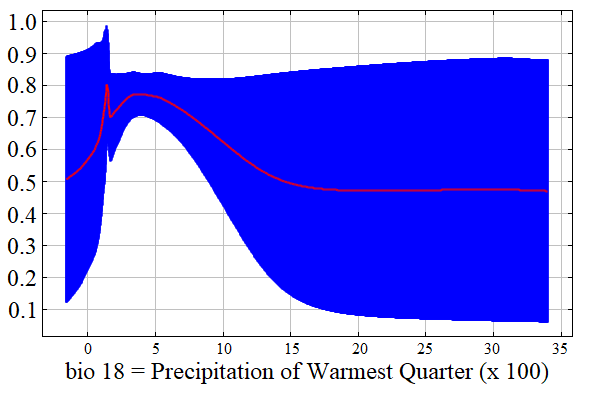 | 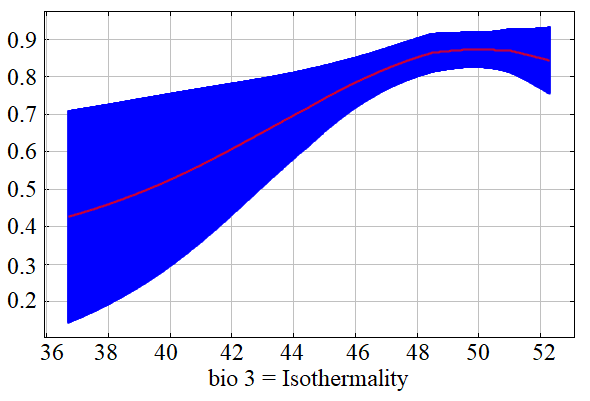 |
| 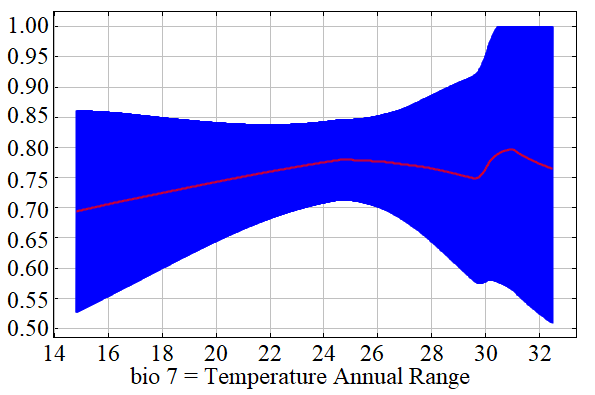 | 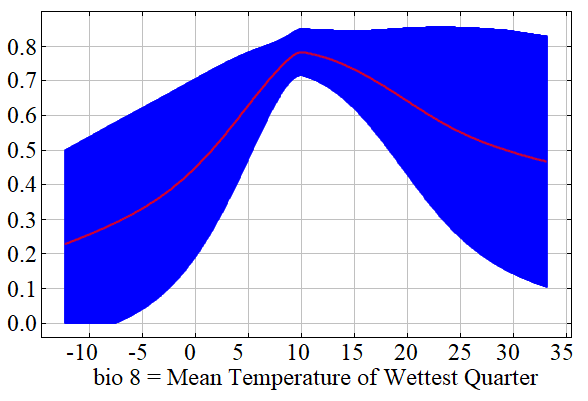 |
| 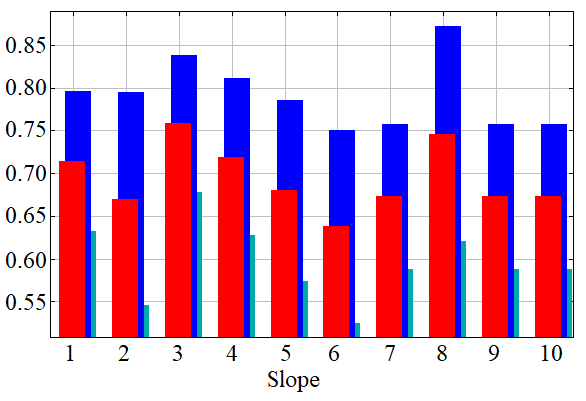 | Y-axes: the probability of presence. Temperatures are expressed in °C and precipitation in mm. Aspects are 1 (North), 2 (North–East), 3 (East), 4 (South–East), 5 (South), 6 (South–West), 7 (West), and 8 (North–West). Slope: 1(1°-8°), 2(8°-13°), 3(13°-18°), 4(18°-23°), 5(23°-28°), 6(28°-33°), 7(33°-38°), 8(38°-43°), 9(43°-48°), and 10(48°-53°). Red, blue, and teal column colours indicate values for average, maximum, and without variables in aspect and slope. |

Fig. S3b. Response curves for predicting variables in modelling the probability of the presence (logistic model) of *N. jatamansi*. Panels show (a) Elevation, (b) Aspect category, (c) Bio 15: precipitation seasonality, (d) Bio 17: Precipitation of driest quarter, (e) Bio 18: precipitation of warmest quarter, (f) Bio 3: isothermality, (g) Bio 7: annual temperature range, (h) Bio 8: mean temperature of the wettest quarter, and (i) slope category.

Fig. S3c. Percentage contribution of variables in modelling *N. jatamansi* distribution in the Nepalese Himalayas. The variables are explained in Table S2a but also see Fig. S3b above. The distribution of the species is almost equally explained by topographic (50.6%) and climatic factors (49.4%), with elevation being dominant (37.8%), followed by mean temperature of the wettest quarter (Bio 8, 33.7%), aspect (8.4%), isothermality (Bio 3, 6.4%), and slope (4.4%). The estimated probability of occurrence was highest around 4000–4600 m, with slightly higher probabilities on northeast-facing slopes of 38–43˚. Climatically, the probability of occurrence was highest in areas with a mean temperature of around 10˚ C in the wettest quarter.

**Supplementary Information 4.** Table S4. Estimated area of habitats suitable for *N. jatamansi* per province and district in the Nepalese Himalayas

Note: Districts in bold are trade case districts; district area data from Central Bureau of Statistics (Statistical year book of Nepal 2019, Kathmandu); adjusted suitability area is the sum of the area in each suitability class using the low-end probabilities (25% of low suitability area, 50% of medium, 75% of high) for each district

**Supplementary Information 5.** Overview of ecological parameter estimates for sustainability assessment of *N. jatamansi*

The literature used to identify the parameter estimates required to assess the sustainability of commercial harvesting (Eq. 1) is provided in Table S5. Many related studies exist but do not address the parameters, e.g. because they only report rhizome stock per m^2^ based on a limited number of plots, or the applied methods are unclear. As rhizome biomass production may vary with altitude and location (39), estimating stock and harvest yield at the province level appeared preferable. However, as the extant work is limited, we used the same estimates throughout the country.

Table S5. Overview of parameter estimates for sustainability assessment of *N. jatamansi* in Nepal

| Parameter | Estimate | Comment |
| --- | --- | --- |
| Adjustment factor (*A*) | 0.46 | There is only one study (40) reducing suitable habitat to effective habitat (46%), based on participatory mapping and ground-truthing in eight Community Forest User Groups in Jumla and Mugu districts (covering 10,088 ha). In Humla, the effective forest area in community forestry (reducing for rocky areas) was estimated at 59% (41) |
| Growing stock (*S*, air-dry kg/ha) | 141 | S ranged from 86-187 with weighted means of 141 and 165 in Jumla and Mugu sites (40). Older inventories reported average stock levels of 1019 kg/ha and 487 kg/ha in Humla District (41, 42) and 682 kg/ha in Manang District (28). Rhizome biomass production varies with altitude and location (39) |
| Yield: share of *S* harvestable per year (*Y*) | 10-100% | Scenario 1 - Conservative: harvest 10% as that is the limit in sensitive rocky outcrop habitats (43)  Scenario 2 - Common: harvest 25% as that is possible in the more common meadow habitat (43)  Scenario 3 – New harvest: harvest 100% with the replanting of the upper plant parts and 2 cm of the rhizome (44) |
| Rotation factor (*R*) | 0.20 | Five years between harvests regardless of habitat (43). This is supported elsewhere (42) recommending five years between harvests as economically optimal (no further significant gain in yields per unit area) |

**Supplementary Information 6**. Table S6. Province and district-level estimates of *N. jatamansi* trade in Nepal (kg air-dried rhizomes) in 1997-98 and 2014-15

Notes:

1. We interviewed the entire population of central wholesalers in Nepal and found no instance of direct purchase of *N. jatamansi* rhizomes from harvesters; hence central wholesalers were not included in the above table.
2. The majority of processors were located in larger cities (45) and, while they purchased directly from harvesters to some extent, we were unable to connect their supplies to individual districts of origin. Here we assigned processor purchases to districts of origin using each district's relative share of the national adjusted suitable production area.
3. Trade generalisation was done by extrapolating from case districts (in bold in the above table) to (i) the other districts in the same cell, or (ii) the nearest neighbour in the same development region when a district had *N. jatamansi* suitable habitat but the case district did not. It was assumed that the quality of production areas and harvesting pressure were similar within cells (and nearest neighbours when relevant). This is supported by the MaxEnt modelling showing that habitat suitability declines from East to West (Fig. 2).
4. Following the same cell/nearest neighbour principle, the district generalisation took place from: Taplejung to Panchthar, Sankhuwasabha, and Solukhumbu; Dolakha to Rasuwa, Sindhupalchok, and Ramechap; Gorkha/Mustang to Dhading, Baglung, Kaski, Lamjung, Manang, and Myagdi; Humla to Dailekh, Dolpa, Jajarkot, Jumla, Kalikot, Mugu, West Rukum, East Rukum, and Rolpa; and Darchula to Bajhang and Bajura.
5. The total amount purchased by processors was 200,888 kg in 1997-98 (46), and 353,803 kg in 2014-15 (36), with a share of 81.8% purchased directly from harvesters. For the 1997-98 data, it was assumed that the share purchased directly from harvesters was the same as in 2014-15.

**Supplementary Information 7**. Overview of price developments for air-dried *N. jatamansi* rhizomes in Nepal and India

Figure S7a presents an overview of purchasing prices paid by central wholesalers in Nepal (average of Kathmandu and Nepalgunj) and regional wholesalers in India (average of Delhi, Kolkata, Lucknow, and Tanakpur). Prices follow each other in the two production network locations as expected, with prices in India consistently higher than in Nepal and rising faster (p=6.95*10^-8^), indicating disproportionate value capture by the exporting central wholesalers in Nepal at the cost of upstream traders and harvesters.

Current prices were reported in the original price sources (listed below). Prices reported in Indian rupees (INR) were converted to NPR using the International Monetary Fund's exchange rates (<https://www.imf.org/external/np/fin/data/param_rms_mth.aspx>). All prices were adjusted to February 2019 prices using the Consumer Price Index, then converted to USD using the USD-Nepalese rupees (NPR) exchange rate from February 2019 (<https://www.ceicdata.com/en/indicator/nepal/exchange-rate-against-usd>).

Nepal prices were obtained and used as follows:

- Most prices were from the non-timber forest product price list maintained by the Asia Network for Sustainable Agriculture and Bioresources (ANSAB), <https://ansab.org.np/sub/ntfps-price-list>. This list is incomplete for *N. jatamansi* air-dry rhizomes and was hence supplemented with other available prices as below. The latest ANSAB data is from January 2020.
- Regarding the few available older prices in 2002-2005 (from ANSAB): in 2002, the graphed price is an average of the June and December price of the two markets; in 2004, it is an average of the two markets’ February and December prices; in 2003 and 2005 it is the average July and November prices.
- The Nepalese price in 2006 is an average of the prices reported from Rasuwa District (33) and the price reported by (47).
- The price in 2007 is from (48).
- It has not been possible to locate prices for 2008 and 2009.
- September and December 2011 prices are based on the International Trade Centre's (ITC's) quarterly market news service on medicinal plants and extracts (e.g. <http://www.intracen.org/uploadedFiles/intracenorg/Content/Exporters/Market_Data_and_Information/Market_information/Market_Insider/Medicinal_plants/Medicinal%20Plants%20December%202011.pdf>). Where prices in ITC's quarterly reports differed from the ANSAB prices, the latter was used.
- Between 2010 and 2019, prices were only graphed when data on both markets were available. Due to missing prices from Kathmandu, especially during the end of 2017 and 2018, there are several missing points.

India prices were obtained and used as follows:

- Most prices were from the non-timber forest product price list maintained by ANSAB, <https://ansab.org.np/sub/ntfps-price-list>. This list is incomplete for *N. jatamansi* air-dry rhizomes and was hence supplemented with other available prices as below.
- Prices for May and December 2016, January 2017, and October 2018 are from the National Medicinal Plant Board of India (<https://www.nmpb.nic.in/content/market-price-medicinal-plants>).
- The price in 2006 is based on the prices reported by (49, 50).
- The price in 2008 is based on the price from Tanakpur (51).
- It has not been possible to locate prices for the Indian market for 2007 and 2009.
- Between 2010 and 2019, prices were only graphed when data from at least two markets were available.

As Indian markets vary in distance from Nepal, we would expect spatial arbitrage. Figure S7b indicates this is the case, with prices in Tanakpur (closest to Nepal) always the lowest and prices in Kolkata (farthest from Nepal) always the highest.

Fig. S7a. Central wholesaler purchasing prices in Nepal (averages from Nepalgunj and Kathmandu) and regional wholesaler prices in India (averages of Delhi, Kolkata, Lucknow, and Tanakpur) for air-dried *N. jatamansi* rhizomes, 2002-2020, adjusted for inflation, all values in February 2019 prices. Major price changes are indicated with arrows 1-5; actors are unable to identify the causes of these changes.


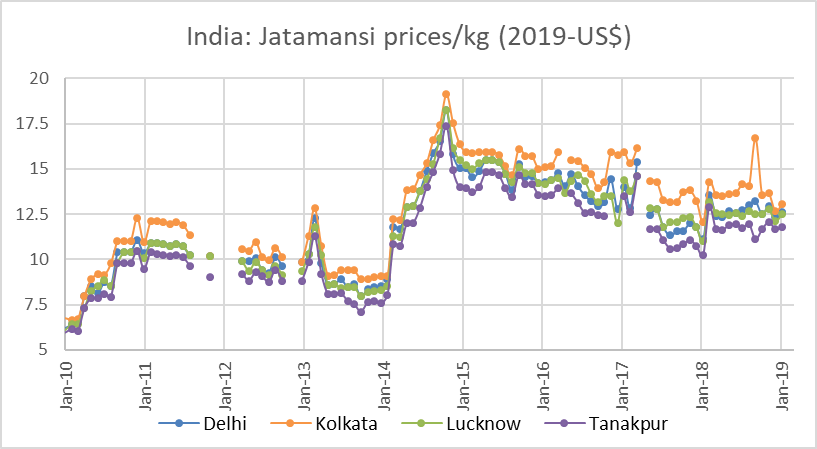


Fig. S7b. Regional wholesaler purchasing prices in Delhi, Kolkata, Lucknow, and Tanakpur for air-dried *N. jatamansi* rhizomes, 2010-2018, adjusted for inflation, all values in February 2019 NPR prices.

**Supplementary Information 8**. Table S8. District, province, and national level sustainability estimates for the trade in air-dried rhizomes of *N. jatamansi* in 1997-98 and 2014-15, Nepal (kg)

Notes: Annual allowable harvest (AAH) rates are from DFSC (undated: National Quota Fixation for Jatamansi (Nardostachys jatamansi DC) Ensuring Sustainable Management and Conservation in Nepal. Department of Forests and Soil Conservation, Kathmandu). Comparing the species distribution with the AAH data shows two differences. First, quotas have been assigned for three districts (50, 5, and 3 t from Dailekh, Doti, and Pyuthan districts) where the species does not occur according to our model. This indicates that some quotas are assigned based on trader location in the production network rather than harvesting sites. Second, the quota system does not include Panchthar, Sankhuwasabha, Dolakha, Kaski, and Mustang districts with suitable jatamansi habitats.

**Supplementary Information 9.** National, provincial, and district level comparisons of *N. jatamansi* trade volumes (kg): the adjusted suitability approach results vs using the two low-cost proxies: total district area and district forest area

At the national level, the district area generalisation results are the same as the adjusted suitable area estimates (+0.4% and -1.1% difference for 1997-98 and 2014-15), while the forest area generalisation overestimates (9.2% and 34.5%), Table S9a. The average differences at the provincial level for the two observation years are 8.1% (-14 to +41%) and 4.6% (-22 to +29%) for the district area approach and 31.5% (-10 to +236%) and 42.1% (-16 to +277%) for the forest area approach, Table S9b. Both approaches tend to underestimate trade in the core production districts and consistently overestimate trade in marginal production districts, up to 32-fold overestimation, Table S9c.

Table S9a. National, province, and district level *N. jatamansi* air-dried rhizome trade volume comparisons

Note: Districts in bold are trade case districts; the district area data was from the Central Bureau of Statistics (2020: Statistical year book of Nepal 2019. Kathmandu) and the forest area data from DFRS (2018: Forest cover maps of local levels (753) of Nepal. Department of Forest Research and Survey, Kathmandu).

Table S9b. Provincial-level summary of differences for both case years. Top: absolute amounts (kg), Bottom: relatively (%) to the adjusted suitability area estimate

Table S9c. District level differences across core production districts (top five supply districts in 2014-15) and marginal production districts (bottom five)

**Supplementary Information 10**. Table S10. District, province, and national level trade in the air-dried rhizomes of *N. jatamansi* from fiscal years 2008/09 to 2015/16, Nepal (kg), according to Department of Forests records (DoF 2009 to 2016)

| **Province** | **Districts** | **FY 2008/09** | **FY 2009/10** | **FY 2010/11** | **FY 2011/12** | **FY 2012/13** | **FY 2013/14** | **FY 2014/15** | **FY 2015/16** |
| --- | --- | --- | --- | --- | --- | --- | --- | --- | --- |
| Bagmati Province |  |  |  |  |  |  |  |  |  |
|  | Dolakha |  | 300 |  |  |  |  |  |  |
|  | Rasuwa |  | 250 |  |  |  |  |  |  |
|  | Dhading |  |  |  |  | 1306 |  |  |  |
|  | Nuwakot | 200 |  |  |  |  |  |  |  |
| Gandaki Province |  |  |  |  |  |  |  |  |  |
|  | Myagdi |  | 20 | 5 | 125 |  | 400 |  | 1000 |
|  | Lamjung |  |  |  | 2000 | 4248 |  |  | 2050 |
|  | Baglung | 220 |  |  |  | 250 | 10 |  |  |
|  | Gorkha | 4850 | 6325 | 5407 | 1295 | 1289 |  |  |  |
| Lumbini Province |  |  |  |  |  |  |  |  |  |
|  | Rukum | 610 | 223 |  | 1881 |  | 1000 |  | 23600 |
| Karnali Province |  |  |  |  |  |  |  |  |  |
|  | Dolpa | 10003 |  | 898 | 8275 | 16750 | 900 | 36400 | 39000 |
|  | Dailekh | 981 | 514 | 564 | 20 |  | 165 | 230 | 9984 |
|  | Jajarkot |  |  | 2264 |  | 1800 |  | 2300 | 12800 |
|  | Humla |  |  |  |  |  |  |  | 30000 |
|  | Mugu | 4321 |  |  |  |  | 2060 | 38322 |  |
|  | Jumla | 32610 |  | 58375 |  |  | 20974 |  |  |
|  | Surkhet |  |  |  |  | 9013 |  |  |  |
| Sudurpaschim Province |  |  |  |  |  |  |  |  |  |
|  | Bajura |  |  |  |  | 1975 |  |  | 3000 |
|  | Bajhang |  | 500 |  |  |  |  | 2184 |  |
|  | Darchula |  | 400 | 750 |  |  |  |  |  |
| Total | | 53795 | 8532 | 68263 | 13596 | 36631 | 25509 | 79436 | 121434 |

**Supplementary Information 11**. CITES Trade Data analysis for *N. jatamansi* for 1997-2017

*N. jatamansi* was proposed for inclusion in CITES Appendix II by India on two occasions, in 1989 and 1994, before its inclusion in 1997. The original listing was annotated to include whole and sliced roots and parts of roots, excluding manufactured parts or derivatives, such as powders, pills, extracts, tonics, teas, and confectionery. In 2007, this annotation was amended to all parts and derivatives except: (i) seeds and pollen, and (ii) finished products packaged and ready for retail trade. In the examined period, no export quotas were established for *N. jatamansi*, but the management Authority of India banned the export for commercial purposes of all wild-taken specimens of species included in Appendices I, II, and III twice, in 1999 and 2018.

Table S11 presents an overview of all reports of exports and imports for the period 1997-2017. Almost the entire trade was wild harvested, for commercial purposes, and took place through processed products (derivatives). There is a massive discrepancy between exporter and importer reporting. The average total trade reported was 49 kg/yr (importer-reported) and 73500 kg/yr (exporter-reported), and thus much below the estimated annual trade level.

Although listed in Appendix II in 1997, no exporter-reported trade was reported until 2001. Trade reported in 2001 related to 12,500 kg of roots exported by China to Nepal. No exporter reported trade between 2002 and 2007, but figures increased from 2008 (Figure S11). It is presumed that this increase coincided with the previously mentioned annotation amendment. Recent increased exporter reporting indicates a more strict implementation of CITES in Nepal, which is responsible for more than 99% of the reported exports. The large discrepancies between the total quantities of importer and exporter reported trade (1,022 kg vs 1,544,034 kg) could be explained by Nepal (the exporter) reporting large amounts of trade to Bangladesh (9,210 kg), India (1,038,553 kg), and Pakistan (477,230 kg), but none of these Parties reporting any imports of *N. jatamansi* commodities.

Re-exports accounted for a tiny proportion of the total trade in *N. jatamansi* commodities. Re-exported trade totalled 107 kg according to exporter-reported quantities and 52 kg according to importer-reported quantities.

Overall, it is hard to say anything firm regarding trade levels, given the vast differences between the importer and exporter-reported quantities. When importer-reported quantities were analysed, European Parties were the leading importers, and imports totalled 1,022 kg. When exporter-reported quantities are analysed, Bangladesh, India, and Pakistan are the leading importers, totalling 1,544,029 kg.

Table S11. Importer and exporter-reported quantities (kg) for trade in *N. jatamansi* from 1997-2017 in the CITES Trade Database by source, purpose, and commodity type.

|  | **Exporter reported** | **Importer reported** |  |
| --- | --- | --- | --- |
| *Source* | | | |
| Artificially propagated | 0 | 25 |  |
| Confiscated/seized specimens | 0 | 1 |  |
| Wild sourced | 1,544,092 | 996 |  |
| Not reported | 5 | 0 |  |
| *Purpose* | | | |
| Educational | 1 | 0 |  |
| Commercial | 1,544,091 | 1,022 |  |
| Not reported | 5 | 0 |  |
| *Commodity* | | | |
| Derivatives | 1,330,915 | 0 |  |
| Extract | 0 | 15 |  |
| Medicine | 0 | 1 |  |
| Oil | 39,704 | 1,006 |  |
| Roots | 173,479 | 0 |  |

|  |
| --- |
|  |

Figure S11. Annual trade volumes (kg) of *N. jatamansi* per nation, based on exporter-reported quantities (panel a) and importer-reported quantities (panel b)

**Supplementary Information 12.** Fieldwork districts, physiographic zones, and development regions

The trade survey was conducted, in both 1997-98 and 2014-15, in 15 case districts: one in each cell created by the intersection of the three physiographic zones (high mountains, middle hills, Terai) and the five development regions (Far-Western, Mid-Western, Western, Central, Eastern). Districts were chosen randomly while not allowing two districts in the same physiographic zone to be adjacent. An overview of the 1997-98 approach is provided by (52) and the 2014-15 approach by (53). The districts are listed in Table S12. An overview of the field district locations is presented in Figure S12.

Table S12. Distribution of the 15 case districts included in the trader survey across physiographic zones and development regions.

|  | High mountains | Middle hills | Terai |
| --- | --- | --- | --- |
| Far-Western | Darchula | Baitadi | Kailali |
| Mid-Western | Humla | Surkhet | Dang |
| Western | Gorkha^1^ | Palpa | Rupandehi |
| Central | Dolakha | Nuwakot | Rautahat |
| Eastern | Taplejung | Udayapur | Morang |

^1^ The 1997-98 survey found Mustang District with a low degree of representativeness of its cell (High mountains in the Western Development Region); hence, in the 2014-15 survey, this district was replaced by Gorkha District.


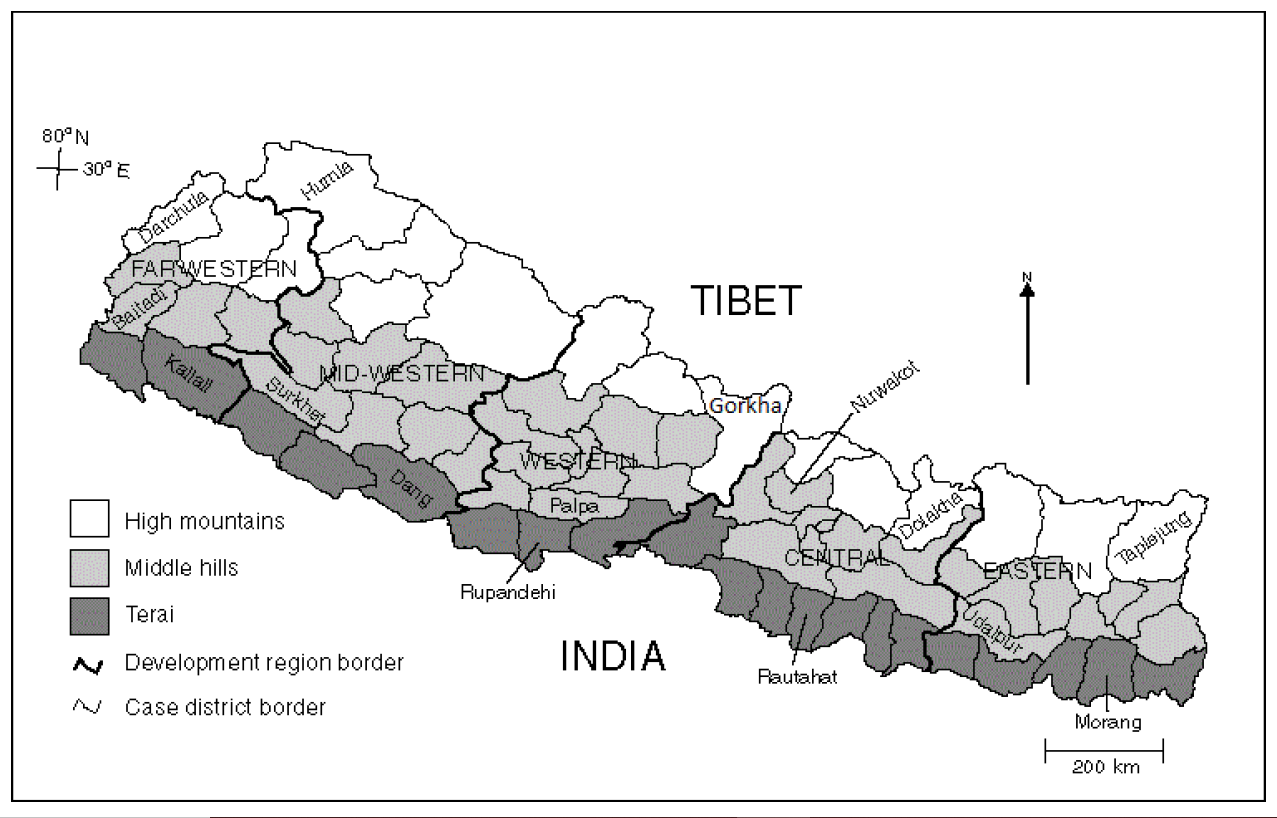


Fig. S12. Map of Nepal showing the five development regions, three physiographic zones, the 15 cells (created by the five ecological zones and three physiographic zones), and the 15 case districts (53)

**References**

1. T. Mulliken, P. Crofton, Review of the status, harvest, trade and management of seven Asian CITES-listed medicinal and aromatic plant species. *BfN – Skripten* 227 (2008).
2. Y. Roskov, L. Abucay, T. Orrell, D. Nicolson, N. Bailly, P. M. Kirk, T. Bourgoin, R. E. DeWalt, W. Decock, A. DeWever, E. V. Nieukerken, J. Zarucchi, L. Penev, Species 2000 & ITIS catalogue of life, 9 September 2019. [www.catalogueoflife.org/col](http://www.catalogueoflife.org/col).
3. F. Weberling, On the systematics of Nardostachys (Valerianaceae). *Taxon* 24, 443-452 (1975).
4. O. Polunin, J. D. A. Stainton, Flowers of the Himalaya. London: Oxford University Press (1984).
5. K. Gautam, R. Raina, Review of Nardostachys grandiflora: An Important Endangered Medicinal and Aromatic Plant of Western Himalaya. *Forest Products Journal* 63(1-2), 67-71 (2013).
6. N. Dhiman, A. Bhattacharya, Nardostachys jatamansi (D. Don) DC. – challenges and opportunities of harnessing the untapped medicinal plant from the Himalayas. *Journal of Ethnopharmacology* 246, 112211 (2020).
7. A. B. Cunningham, J. A. Brinckmann, X. Yang, J. He, Introduction to the special issue: Saving plants, saving lives: Trade, sustainable harvest and conservation of traditional medicinals in Asia. *Journal of Ethnopharmacology* 229, 288-292 (2019).
8. A. Hinsley, E. J. Milner-Gulland, R. Cooney, A. Timoshyna, X. Ruan, T. M. Lee, Building sustainability into the Belt and Road Initiative's Traditional Chinese Medicine trade. *Nature Sustainability* 3, 96–100 (2020).
9. D. Pyakurel, I. B. Sharma, C. Smith-Hall, Patterns of change: the dynamics of medicinal plant trade in far-western Nepal. *Journal Ethnopharmacology* 224, 323-334 (2018).
10. H. Kaur, M. M. Lekhak, S. Chahal, U. Goutam, P. Jha, D. Naidoo, S. J. Ochatt, V. Kumar, Nardostachys jatamansi (D. Don) DC.: an invaluable and constantly dwindling resource of the Himalayas. *South African Journal of Botany* 135, 1-16 (2020).
11. T. Rehman, S. Ahmad, Nardostachys chinensis Batalin: a review of traditional uses, phytochemistry, and pharmacology. *Phytotherapy Research* 33, 2622-2648 (2018).
12. F. Weberling, Monographie der gattung Nardostachys DC. (Valerianaceae). *Botanisches Jahrbücher* 99, 189–221 (1978).
13. GBIF, GBIF Occurrence Download <https://doi.org/10.15468/dl.de9ncu> (2019).
14. D. Pyakurel, C. Smith-Hall, I. B. Sharma, S. K. Ghimire, Trade and conservation of Nepalese medicinal and aromatic plants, fungi, and lichen. *Economic Botany* 73, 505–521 (2019).
15. T. A. Mulliken, Implementing CITES for Himalayan medicinal plants Nardostachys grandiflora and Picrorhiza kurroa. *TRAFFIC Bulletin* 19(2), 63-72 (2000).
16. S. K. Ghimire, D. Pyakurel, B. K. Nepal, I. B. Sapkota, R. Rai-Parajuli, B. R. Oli, A manual of NTFPs of Nepal Himalaya (In Nepali). WWF, Kathmandu (2008).
17. S. K. Ghimire, Y. Aumeeruddy-Thomas, “Approach to in situ conservation of threatened Himalayan medicinal plants: a case study from Shey-Phoksundo National Park, Dolpo” in Himalayan Medicinal and Aromatic Plants, Balancing Use and Conservation, Y. Aumeeruddy-Thomas, M. Karki, K. Gurung, D. Parajuli, Eds. (Ministry of Forests and Soil Conservation, 2005), pp. 209-234.
18. G. Amatya, V. Sthapit, V, A note on Nardostachys jatamansi. *Journal of Herbs, Spices & Medicinal Plants* 2(2), 39-47 (1994).
19. B. Vaidya, Livelihoods and sustainability aspects of nontimber forest products in Gorkha district, Nepal. MSc Dissertation, Agricultural University of Norway (2002).
20. D. Ved, D. Saha, K. Ravikumar, K. Haridasan, Nardostachys jatamansi. The IUCN Red List of Threatened Species, e.T50126627A50131395 (2015).
21. DPR, Prioritised Medicinal Plants for Economic Development in Nepal (Nepal ko Aarthik Bikaska lagi Prathamikata Prapta Jadibutiharu). Department of Plant Resources, Kathmandu (2006).
22. MFSC, Forest sector strategy (2016-25). Ministry of Forests and Soil Conservation, Kathmandu (2016).
23. C. Kirkpatrick, Account of the Kingdom of Nepaul, being the substance of observations made during a mission to that country in the year 1793. Asian Educational Services, New Delhi. Reprint 1986 (1811).
24. C. S. Olsen, Valuation of commercial central Himalayan medicinal plants. *Ambio* 34(8), 607-610 (2005).
25. K. R. Amatya, MAPs trade and promotion. *Plant Resources* 22, 104-117 (2003).
26. DFSC, National Quota Fixation for Jatamansi (Nardostachys jatamansi DC) Ensuring Sustainable Management and Conservation in Nepal. Department of Forests and Soil Conservation, Kathmandu (undated).
27. K. K. Shrestha, S. K. Ghimire, Diversity, Ethnobotany and Conservation Strategy of Some Potential Medicinal and Aromatic Plants of Taplejung in Tamur Valley. Asia Network for Small-scale Agricultural Biotechnologies (1996).
28. D. B. Chhetri, Diversity of Medicinal and Aromatic Plants in Manang, Central Nepal with Emphasis on Ecology and Essential Oil Variation of Jatamansi (Nardostachys grandiflora DC.). MSc Dissertation, Central Department of Botany, Tribhuvan University, Kathmandu (1999).
29. R. Roy, Contribution of NTFPs [Non-Timber Forest Products] to Livelihood in Upper Humla, Nepal. Asian Institute of Technology (2010).
30. DoF, Hamro Ban. Department of Forests, Kathmandu (2009 – 2016).
31. D. M. Edwards, The marketing of non-timber forest products from the Himalayas: The trade between east Nepal and India. *Rural Development Forestry Network Paper* 15b (1993).
32. C. S. Olsen, F. Helles, Medicinal plants, markets, and margins in the Nepal Himalaya: Trouble in paradise. *Mountain Research and Development* 17(4), 363–374 (1997).
33. K. Humagain, K. K. Shrestha, Medicinal plants in Rasuwa district, central Nepal: Trade and livelihood. *Botanica Orientalis* 6, 39–46 (2009).
34. N. K. Bhattarai, “Medicinal and aromatic plants of Nepal” in The Role of Bamboo, Rattan and Medicinal Plants in Mountain Development, M. Karki, A. N. Rao, R. Rao, J. T. Williams, Eds. (International Network for Bamboo and Rattan, 1997), pp. 162-173.
35. IRG, Role of natural products in resource management, poverty alleviation, and good governance: A case study of jatamansi and wintergreen value chain in Nepal. International Resource Groups, Washington DC (2006).
36. F. Caporale, J. Mateo-Martín, M. F. Usman, C. Smith-Hall, Plant-based sustainable development— the expansion and anatomy of the medicinal plant secondary processing sector in Nepal. *Sustainability* 12(14), 5575 (2020).
37. DFSC, CITES. https://www.dofsc.gov.np/page/cites/en (accessed 7th July 2022).
38. ANSAB, Brief Jatamansi profile. Asia Network for Sustainable Agriculture and Bioresources. Kathmandu (2019).
39. H. O. Larsen, Commercial medicinal plant extraction in the hills of Nepal: local management system and ecological sustainability. *Environmental Management* 29(1), 88–101 (2002).
40. ANSAB, Technical report on resources inventory of jatamansi (Nardostachys jatamansi DC.) in the targeted community forests of Jumla and Humla. Asia Network for Sustainable Agriculture and Bioresources, Kathmandu (2020).
41. ANSAB, Inventory of non-timber forest products at Mimi, Melchham and Darma VDCs, Humla, Nepal. Asia Network for Sustainable Agriculture and Bioresources, Kathmandu (2002).
42. ANSAB, Monitoring the effects of community based conservation and commercial utilisation of natural products on biodiversity in Humla, Nepal. Asia Network for Sustainable Agriculture and Bioresources, Kathmandu (1999).
43. S. K. Ghimire, O. Gimenez, R. Pradel, D. McKey, Y. Aumeeruddy-Thomas, Demographic variation and population variability in a threatened Himalayan medicinal and aromatic herb Nardostachys grandiflora: matrix modelling of harvesting effects in two contrasting habitats. *Journal of Applied Ecology* 45(1), 41-51 (2008).
44. H. O. Larsen, Impact of replanting on regeneration of the medicinal plant Nardostachys grandiflora DC. (Valerianaceae). *Economic Botany* 59(3), 213-220 (2005).
45. A. Chapagain, G. Kafle, A. K. Das, F. Caporale, J. Mateo-Martin, F. Usman, M. Pouliot, C. Smith-Hall, A population list of medicinal plant processing enterprises in Nepal. IFRO Documentation Series 2019/3, University of Copenhagen (2019).
46. C. S. Olsen, Trade and conservation of Himalayan medicinal plants: Nardostachys grandiflora DC. and Neopicrorhiza scrophulariiflora (Pennell) Hong. *Biological Conservation* 125, 505-514 (2005).
47. S. K. Ghimire, I. Sapkota, B. Oli, R. Parajuli-Rai, Non-timber forest products of Nepal Himalaya: Database of some important species found in the mountain protected areas and surrounding regions. WWF Nepal, Kathmandu (2008).
48. WWF, Gift of the Himalayas. WWF Nepal, Kathmandu. Available at <http://www.wwfnepal.org/?118460/Gift-of-the-Himalayas-high-value-plants-and-NTFPs> (2007).
49. Samant, S. Pant, M. Singh, M. Lal, A. Singh, A. Sharma, S. Bhandari, Medicinal plants in Himachal Pradesh, north western Himalaya, India. *The International Journal of Biodiversity Science and Management* 3, 234-251 (2007).
50. J. S. Butola, H. K. Badola, Threatened Himalayan medicinal plants and their conservation in Himachal Pradesh. *Journal of Tropical Medicinal Plants* 9, 125-142 (2008).
51. D. Arya, G. Joshi, L. M. Tiwari, Status and trade of crude drug in Uttarakhand. *Journal of Medicinal Plants Research* 6, 3434-3444 (2012).
52. N. K. Bhattarai, C. S. Olsen, “Towards a generic framework for investigating national importance of medicinal plant trade” in Community based NTFP management, S. M. Amatya, Ed. (South and East Asian Countries NTFP Network, 2000), pp. 336-348.
53. C. Smith-Hall, M. Pouliot, D. Pyakurel, N. Fold, A. Chapagain, S. Ghimire, H. Meilby, L. Kmoch, D. J. Chapagain, A. Das, H. Jun, K. Nepal, M. R. Poudeyal, G. Kafle, H. O. Larsen, Data collection instruments and procedures for investigating national-level trade in medicinal and aromatic plants. IFRO Documentation 2018/2, University of Copenhagen (2018).
